# PEGylation of Metal Oxide Nanoparticles Modulates Neutrophil Extracellular Trap Formation

**DOI:** 10.3390/bios12020123

**Published:** 2022-02-16

**Authors:** Hunter T. Snoderly, Kasey A. Freshwater, Celia Martinez de la Torre, Dhruvi M. Panchal, Jenna N. Vito, Margaret F. Bennewitz

**Affiliations:** Department of Chemical and Biomedical Engineering, West Virginia University, Morgantown, WV 26506, USA; htsnoderly@mix.wvu.edu (H.T.S.); kaf0005@mix.wvu.edu (K.A.F.); cemartinezdelatorre@mix.wvu.edu (C.M.d.l.T.); dp00016@mix.wvu.edu (D.M.P.); jnv0006@mix.wvu.edu (J.N.V.)

**Keywords:** manganese oxide, iron oxide, nanoparticles, gadolinium chelates, contrast agents, magnetic resonance imaging, neutrophils, neutrophil extracellular traps

## Abstract

Novel metal oxide nanoparticle (NP) contrast agents may offer safety and functionality advantages over conventional gadolinium-based contrast agents (GBCAs) for cancer diagnosis by magnetic resonance imaging. However, little is known about the behavior of metal oxide NPs, or of their effect, upon coming into contact with the innate immune system. As neutrophils are the body’s first line of defense, we sought to understand how manganese oxide and iron oxide NPs impact leukocyte functionality. Specifically, we evaluated whether contrast agents caused neutrophils to release web-like fibers of DNA known as neutrophil extracellular traps (NETs), which are known to enhance metastasis and thrombosis in cancer patients. Murine neutrophils were treated with GBCA, bare manganese oxide or iron oxide NPs, or poly(lactic-co-glycolic acid) (PLGA)-coated metal oxide NPs with different incorporated levels of poly(ethylene glycol) (PEG). Manganese oxide NPs elicited the highest NETosis rates and had enhanced neutrophil uptake properties compared to iron oxide NPs. Interestingly, NPs with low levels of PEGylation produced more NETs than those with higher PEGylation. Despite generating a low rate of NETosis, GBCA altered neutrophil cytokine expression more than NP treatments. This study is the first to investigate whether manganese oxide NPs and GBCAs modulate NETosis and reveals that contrast agents may have unintended off-target effects which warrant further investigation.

## 1. Introduction

Cancers are diagnosed using a variety of screening methods, such as mammography, ultrasound, and magnetic resonance imaging (MRI). Although mammography is the current gold standard for breast imaging, it leads to inaccurate diagnoses, especially in younger patients where dense breasts can obscure cancer [1,2,3]. Therefore, ultrasound and MRI are used as secondary imaging tools in cases where mammography is suboptimal to visualize additional breast cancers [4,5,6]. Neither ultrasound or MRI have ionizing radiation, but ultrasound suffers from a limited depth of penetration which can miss some malignancies deeper in the breast [7,8]. In contrast, MRI acquires full 3D images of the breast with high spatial resolution and excellent soft tissue contrast that is capable of distinguishing between fat, muscle, organs, and tumors. However, MRI without additional contrast agents, suffers from exceedingly poor sensitivity [9]. To overcome this, contrast agents alter the relaxation rates of protons within water molecules in the body, thereby enhancing the magnitude of the signal in the tissues and organs where the contrast agent is incorporated [10].

Several types of contrast agents, including magnetic chelates and nanoparticles (NPs), have been created in recent decades to improve MRI sensitivity. Gadolinium (Gd) based contrast agents (GBCAs) and metal oxide NPs have both been used clinically to enhance the MRI-based tumor characterization [11]. While GBCAs are more commonly used than metal oxide contrast agents, GBCAs have recently fallen under increased regulatory scrutiny. The FDA has raised concerns around the safety of GBCAs due to reports of nephrogenic systemic fibrosis in renally challenged patients [12], gadolinium accumulation in the brain and other tissues following multiple contrast agent administrations [13,14,15], and in vitro neurotoxicity [16,17]. Additionally, one study suggested prenatal Gd exposure enhanced the risk of the child developing an inflammatory condition within 4 years after birth [18]. Due to these concerns and the favorable magnetic properties of metal oxides, metal oxide NPs have emerged as attractive possible alternatives to traditional GBCAs. Iron oxide (Fe_2_O_3_ or Fe_3_O_4_) and manganese oxide (MnO, MnO_2_, or Mn_3_O_4_) are two such NP formulations, with iron oxide providing T_2_ or T_2_ *-weighted contrast and manganese oxide providing T_1_-weighted contrast [19].

A key advantage of NPs lies in their customizability; different physicochemical properties of NPs can be altered to achieve the desired goal including size [20,21,22,23,24], shape [25,26,27], charge [24,28,29], type of contrast agent [19,30,31], polymer or lipid encapsulation [19,31,32,33,34], surface attachments (e.g., stealth polymers) [35], and the presence of targeting agents [36,37]. However, each modification alters how a given NP formulation will interact with blood cells as well as which tissues it accumulates in before clearance. Upon injection, NPs encounter a series of obstacles that influence their delivery to the target tissue depending on the particle formulation. As soon as NPs enter a patient’s body, they immediately interact with the circulation, resulting in the formation of a protein corona [38,39,40,41], the composition of which varies depending on the surface properties of the NPs [42,43,44]. In turn, this protein corona influences how well phagocytes recognize and internalize NPs. Tissue-resident phagocytes, such as macrophages, have been most studied in relation to NPs, as phagocytosis by these cells ultimately reduces the effective NP dose to the desired imaging areas [38,45]. However, neutrophils, which are circulating phagocytes and the most abundant immune cells in the body, also play a major role in NP clearance [46,47,48]. One NP surface modification technique used to reduce macrophage uptake is the attachment of poly(ethylene glycol) (PEG) chains onto NP surfaces. PEGylation is thought to decrease protein adsorption, thereby reducing phagocyte uptake and increasing the NP circulation time [49,50,51]. However, some studies have observed that PEGylation may have the opposite effect in neutrophils [52], suggesting that NP formulations designed solely with macrophages in mind are non-optimal upon initial delivery when neutrophils are the dominant phagocytes the NPs encounter. Additionally, as neutrophils are key mediators of inflammation and are the body’s first line of immunity, it is vital to consider whether NP uptake by neutrophils results in off-target effects that undermine the intended diagnostic or therapeutic utility of NPs.

When stimulated by inflammatory or pathogenic stimuli, neutrophil nuclei decondense and are exported from the cell in a fibrous structure composed of DNA, histones, and granular content [53,54]. This structure is known as a neutrophil extracellular trap (NET); NETosis refers to the process by which NETs are formed and released. The evolutionary purpose of NETs is predominantly antimicrobial defense, as NETs physically entrap bacteria, facilitating their phagocytosis or killing them directly through proteolytic enzymes decorating the fibers. However, inappropriate NETosis also occurs in the context of inflammation. Indeed, NETs have been observed to drive pathology in inflammatory diseases as diverse as type I diabetes [55], gout [56,57], cystic fibrosis [58], and ulcerative colitis [59]. As cancer is itself an inflammatory disease, it is unsurprising that NETosis also plays a role in tumor progression and spread. Mounting evidence suggests that NETs act as agents of metastasis by trapping circulating tumor cells [60,61,62,63,64]. As such, it is critical to ensure that current and emerging diagnostic tools do not enhance NETosis in a manner that could ultimately worsen cancer patients’ disease progression.

NETosis affects several cancers such as small bowel cancer, breast cancer, colorectal cancer, and lung cancer [53]. In recent years, researchers have found evidence that NETs enhance cancer progression in numerous ways. Higher levels of NET markers, such as cell-free double stranded DNA (dsDNA) [65], citrullinated histone 3 [66], neutrophil elastase [67], or myeloperoxidase [68], are associated with increased tumor cell proliferation and ultimately increased mortality in cancer patients [53,60,61,69]. NET formation also enhances thrombogenesis [70,71]. Since venous thromboembolism (VTE) is relatively common in many cancer patients [72,73], particularly those in later stages of disease, pro-thrombotic effects of diagnostic agents should be mitigated as much as possible. Therefore, it is critical that diagnostic tools such as MRI contrast agents do not enhance NETosis, even if this effect is transient. Higher rates of NETosis throughout the body represent a key opportunity for the establishment of metastatic lesions [61,74,75].

Little is known about how nanomaterials impact NETosis, much less whether specifically metal oxide NPs and GBCAs enhance NETosis. How the physiochemical attributes of NPs modulate neutrophil behavior has been studied in gold, silver, and iron oxide NPs [76,77,78,79,80]. Manganese oxide NPs have not been studied specifically in regards to NETosis, but inhaled Mn_3_O_4_ is known to cause respiratory inflammation; in fact, one study found that Mn_3_O_4_ induced increases of oxidative stress in human alveolar macrophages of up to 700%, as well as more modest increases in oxidative stress in human airway epithelial cells [81]. Therefore, it is reasonable to conclude that manganese oxide NPs may generate oxidative stress in neutrophils as well to contribute to inflammation and possibly NET formation.

Meanwhile, gold NPs (AuNPs) are known to promote NETosis by altering neutrophil surface charge density [79]. Bartneck et al. [76] treated human neutrophils with AuNPs of varying shapes and/or surface chemistries. NETosis was observed 15 min post-AuNP-exposure. Interestingly, the AuNP surface chemistry had a negligible impact on NET formation; however, released NETs tended to capture more positively charged AuNPs within their fibrous networks over time compared to negatively charged AuNPs. Elevated entrapment of positively charged particles was thought to be due to electrostatic attraction of the cationic AuNPs with the anionic NETs containing negatively charged DNA [76]. Hwang et al. have also shown that the NP surface charge modulates neutrophil uptake; liposomes treated with cationic surfactants enhanced neutrophil endocytosis, cytotoxicity, and subsequent NET release [82]. Conversely, Yang et al. [83] recently observed that negatively charged AuNPs synergistically enhanced NET formation in the presence of lipopolysaccharide, a bacterial membrane component and a classical NET stimulant. This effect was observed to be size-dependent, with smaller NPs being taken up more efficiently by neutrophils.

The capacity of NPs to cause NETosis has also been investigated in silver NPs (AgNPs), particularly in the context of whether AgNP size or concentration influences NET formation [77,79,80]. Human neutrophils were exposed to 5 nm AgNPs and 100 nm AgNPs at varying concentrations of 0 µg/mL to 2 µg/mL for 4 h. Starting at a concentration of 1.2 µg/mL, the 5 nm AgNPs induced significant NETosis which increased with higher NP concentrations; however, the 100 nm AgNPs did not induce NETosis regardless of concentration [77]. Another study reported that 15 nm AgNPs induced neutrophil necrosis, increased oxidative stress, and facilitated the release of interleukin-1β, which itself can stimulate further NETosis and enhance neutrophil recruitment [79,80,84].

Prior to this study, iron oxide was the only metal oxide MRI contrast agent that had been studied in the context of NETosis specifically. Bilyy et al. [78] examined iron oxide NPs with either lauric acid-treated or untreated surfaces. NPs were then exposed to serum albumin to form a corona. Interestingly, when placed within a magnetic field, NPs without an albumin coating formed aggregates which caused NETosis. In vivo, non-coated iron oxide NP aggregates further adhered to NETs and promoted vessel occlusion in rabbit veins which persisted for up to 3 days. While the stabilized NPs that remained dispersed in solution were not observed to cause NETosis, this raises the possibility that larger particles, or particles that form aggregates, could promote NETosis. Given the limited research into metal oxide NPs and NET formation, additional study is necessary to determine whether and to what extent the design of novel MRI contrast agents should account for an increased risk of NETosis.

In this study, we sought to determine: (1) whether NETosis is primarily dependent on NP metal oxide content or surface coating, and (2) how NETosis resulting from metal oxide NPs compares to NETosis caused by conventional GBCAs. BALB/c murine-derived neutrophils were treated with standardized concentrations of either chelated Gd or poly(lactic-co-glycolic acid) (PLGA)-coated metal oxide contrast agents (either Fe_3_O_4_ NPs or MnO NPs) with varying amounts of PEGylation. Chelated Gd was used as there is a dearth of information as to whether GBCAs induce NETosis; in fact, to the best of our knowledge, we are the first to investigate the impact of manganese oxide NPs and clinically relevant GBCAs on NETosis. We hypothesized that the surface treatment, particularly a higher PEGylation, which has previously been shown to enhance neutrophil phagocytosis [52], would increase NETosis by causing a greater neutrophil uptake, to produce a greater effective metal oxide dose. Additionally, we expected to observe increased NETosis in manganese oxide NP formulations, as Mn_3_O_4_ NPs have been shown to enhance the inflammatory response relative to Fe_2_O_3_ NPs in other cell populations, specifically epithelial cells [85]. While we did observe that neutrophils were more likely to undergo NETosis in the presence of MnO NPs compared to Fe_3_O_4_ NPs or a conventional GBCA, this effect did not linearly increase with PEGylation. Rather, NPs coated with an intermediate quantity of PEG produced the most NETosis for both MnO and Fe_3_O_4_-based NPs. Interestingly, MnO NPs were taken up more efficiently by neutrophils than Fe_3_O_4_ NPs; however, different levels of PEGylation did not appear to significantly impact overall particle uptake. Ultimately, this suggests that both the formulation and surface functionalization of metal oxide NPs can be tweaked in order to modulate the pro-NETotic response. The design of future MRI contrast agents utilizing metal oxide NPs should account for contrast agent-mediated NETosis in order to minimize the potential negative outcomes that NETs promote in the context of cancer.

## 2. Materials and Methods

### 2.1. Chemicals and Biologicals

Manganese (II) acetylacetonate (Mn(AcAc)_2_, technical grade, ≥97%), oleylamine (technical grade, 70%), and poly(vinyl alcohol) (PVA) were purchased from Sigma-Aldrich. Iron (III) acetylacetonate (Fe(AcAc)_3_, technical grade, ≥98%, TCI Chemicals), dibenzyl ether (≥99%, Acros Organics), dichloromethane (99.5% stabilized ACS, BDH Chemicals), Dulbecco’s phosphate-buffered saline (PBS), Hank’s Balanced Salt Solution (HBSS), serum-free RPMI-1640 media, and formaldehyde (16% Ultra-Pure EM Grade, Polysciences, Inc.) were purchased from VWR. Carboxylic acid-terminated, 50:50 poly(D,L-lactide-co-glycolide) (PLGA) (inherent viscosity: 0.15–0.25 dL/g) was obtained from LACTEL Absorbable Polymers and PLGA (7.5 kDa)–PEG (2 kDa)–alkene (ALK) was obtained from Nanosoft Polymers. Ethanol (Decon Labs, Inc.) was obtained internally from West Virginia University’s Environmental Health and Safety Services. Gado-DTPA™ (Gd-DTPA) was purchased from BioPAL. Hydrochloric acid (HCl) TraceMetal™ Grade, Hoescht 33342, CellTracker™ Deep Red, and a Quant-iT™ PicoGreen™ dsDNA Assay Kit were purchased from ThermoFisher Scientific. Osmium tetroxide (OsO_4_) and hexamethyldisilazane (HMDS) were obtained from Electron Microscopy Sciences. An OxiSelect™ In Vitro ROS/RNS Assay Kit and a Proteome Profiler Mouse Cytokine Array Kit, Panel A were purchased from Cell Biolabs and R&D Systems, respectively.

### 2.2. Animals

BALB/c mice (female, 8 weeks old) were purchased from The Jackson Laboratory (Bar Harbor, ME, USA). Mice were housed in standard cages prior to their use at approximately 12 weeks of age. Studies were conducted in accordance with the West Virginia University Institutional Animal Care and Use Committee (Protocol #1805014087_R1).

### 2.3. Metal Oxide Nanoparticle Fabrication

#### 2.3.1. Synthesis of Manganese Oxide (MnO) and Iron Oxide (Fe_3_O_4_) Nanoparticles

NP synthesis and polymer encapsulation were performed under a chemical fume hood. According to established methods, MnO and Fe_3_O_4_ NPs were fabricated via thermal decomposition of Mn(AcAc)_2_, or Fe(AcAc)_3_, respectively [19,86,87]. Briefly, for thermal decomposition, 6 mmol of Mn(AcAc)_2_ for MnO and 3 mmol of Fe(AcAc)_3_ for Fe_3_O_4_ was dissolved in 10 mL of oleylamine and 50 mL of dibenzyl ether. To control the type of oxidation, the reaction was performed under inert conditions using nitrogen (N_2_) gas and was heated using a heating mantle connected to a programmable temperature controller. For MnO NPs, a one-step temperature ramp was used; the Mn(AcAc)_2_ solution was heated to 60 °C over 30 min and was held there for 1 min. Using a 10 °C/min ramp, the solution was then heated to 280 °C, and after 30 min at this temperature, the reaction was completed and allowed to cool to room temperature. For Fe_3_O_4_ NPs, the Fe(AcAc)_3_ solution was heated to 60 °C over 30 min and was held there for 1 min. Using a two-step temperature ramp of 22 °C/min, the solution was first heated to 110 °C and was held there for 1 h to allow for dehydration, which ensures the maximal formation of an Fe_3_O_4_ nucleus, which is required for nanocrystal formation [87]. Following dehydration, the solution was then heated to 300 °C to convert the Fe_3_O_4_ nucleus to Fe_3_O_4_ nanocrystals via the process of nucleation and it was then aged for 1 h to hold the oxidation state and ensure complete reaction. After aging, the reaction was completed and allowed to cool to room temperature. Following cooling, both MnO and Fe_3_O_4_ NPs were precipitated and washed using ethanol/hexane, collected by centrifugation, resuspended in hexane, and dried. The formation of Fe_3_O_4_ was confirmed based on attraction to a magnetic disc.

#### 2.3.2. Polymer Encapsulation of Metal Oxide NPs 

Using a previously established oil-in-water emulsion technique [19], the metal oxide NPs were encapsulated in PLGA with either 0%, 2.5%, or 5% (*w*/*w*) PLGA–PEG co-block polymer with dichloromethane (DCM) as the organic solvent and PVA as the stabilizing agent [19,88]. A 1:2 ratio (*w*/*w*) of metal oxide to polymer was added to the DCM. After the polymer was dissolved, the NP/polymer/solvent suspension was slowly added dropwise to an aqueous 10% (*w*/*v*) PVA solution under vortex. Once the addition was completed, the solution was vortexed for an additional 10 s and then ultrasonicated for 3, 15 s cycles using a Qsonica Sonicator 125 W to develop the single emulsified NPs. Immediately after sonication, the mixture was poured into a 0.3% (*w*/*v*) aqueous PVA solution under moderate stirring for 3 h to allow for the evaporation of the DCM. The resulting encapsulated NPs were collected by centrifugation, washed, and then resuspended in deionized water. The NPs were lyophilized without the use of cryoprotectants and stored at −80 °C until later use. The polymer encapsulated MnO will be referred to as Nano-, Encapsulated Manganese Oxide (NEMO) particles, and the polymer encapsulated Fe_3_O_4_ will be referred to as Nano-, Encapsulated Iron Oxide (NEIO) particles from here on in.

### 2.4. Nanoparticle Characterization

#### 2.4.1. X-ray Diffraction (XRD)

A Panalytical X’Pert Pro X-ray diffractometer equipped with a Cu K-alpha X-ray source operating at 45 kV and 40 mA in the Bragg-Brentano geometry was used to acquire the XRD patterns of bare MnO and Fe_3_O_4_ NPs. A 1D silicon strip X-ray detector was used to capture spectra throughout a 2θ range of 20° to 85° with a step size of 0.033°. X’Pert HighScore Plus software was used to evaluate the collected XRD patterns. The software calculated the composition of our samples by comparing the XRD spectra of our produced bare MnO and Fe_3_O_4_ NPs with the known spectra for MnO/Mn_3_O_4_/Mn_2_O_3_ and Fe_3_O_4_/Fe_2_O_3_, respectively.

#### 2.4.2. Electron Microscopy

Bare metal oxide NP samples were prepared for transmission electron microscopy (TEM), high-resolution TEM (HRTEM), and selected area electron diffraction (SAED) according to previous studies [19,86] and were imaged using a JEOL JEM-2100 transmission electron microscope at 200 kV. The dry-size diameters and d-parameters for the bare metal oxide NPs were obtained using ImageJ software.

Polymer encapsulated NEIO and NEMO particles were characterized with scanning electron microscopy (SEM) for morphology using a Hitachi scanning electron microscope S4700 operated at 5 kV. Metal oxide encapsulation by polymers was confirmed with SEM plus energy-dispersive X-ray spectroscopy (EDS) using an EDAX Team EDS System operated at 15 kV. During SEM-EDS, lower magnifications were used to decrease the risk of the NPs melting due to the strength of the electron beam.

#### 2.4.3. Dynamic Light Scattering (DLS) and Zeta Potential (ζ-Potential)

Hydrodynamic size distributions and ζ-potential for NEMO and NEIO particles suspended in deionized water were measured with a Malvern Zetasizer Nano ZS (Malvern Instruments). Note that for NP populations that were polydisperse, the data processing tool “multiple narrow modes” was used.

#### 2.4.4. Fourier Transform InfraRed Spectroscopy (FTIR)

A DIGILAB FTS 7000 FTIR spectrometer equipped with a GladiATR attenuated total reflectance module from PIKE Technologies was used to obtain FTIR spectra for bare metal oxide NPs, NEIO particles, and NEMO particles.

#### 2.4.5. ThermoGravimetric Analysis (TGA)

TGA measurements of polymer encapsulated NEIO and NEMO particles were performed using an SDT 650 instrument (TA instruments) [89]. In brief, each sample was loaded at room temperature for 120 min while the chamber was flushed with N_2_ gas. To remove excess water, the samples were heated to 105 °C at 10 °C/min and aged for 1 h. After aging, the samples were heated to 500 °C with a 10 °C/min temperature ramp. Immediately after reaching 500 °C, the samples were heated to 800 °C with a 5 °C/min temperature ramp and were held isothermally for 1 h. Throughout the experiment, temperature, heat flow, and weight loss were recorded. By applying the known thermal decomposition temperatures for PLGA (205 °C–280 °C) [88,90] and PLGA-PEG (334.4 °C) [91], MATLAB^®^ was used to calculate the grafting density and subsequently the % PLGA-PEG for each NP sample using the Equation (1) listed below [92,93]:

(1) $$Grafting\;Density=\frac{\frac{W_{PLGA-PEG}}{100-W_{PLGA-PEG}}*\rho_{PLGA}*V_{shell}*N_{A}}{MW_{PLGA-PEG}*SA_{particle}}$$

where *W_PLGA-PEG_* is the weight loss due to the decomposition of PLGA-PEG (%), *ρ_PLGA_* is the density of PLGA (1.2 g/mL [94]), *V_shell_* is the volume of the polymer encapsulation determined as the difference between the bare metal oxide NP size (TEM) and the weighted average of the hydrodynamic size (DLS), *N_A_* is Avogadro’s number, *MW_PLGA-PEG_* is the molecular weight of PLGA-PEG (9.5 kDa), and *SA_particle_* is the surface area of the polymer-encapsulated NEIO and NEMO particles.

#### 2.4.6. Metal Content and Encapsulation Efficiency Assessment

As described in our previous research, the metal content and the encapsulation efficiency (EE) percentage of each type of NEMO or NEIO particles was measured by fully dissolving ~10 mg of NPs in 150 µL of HCl trace metal grade, followed by the analysis of metal content by inductively coupled plasma-optical emission spectrometry (ICP-OES) [19,86].

### 2.5. Neutrophil Extracellular Trap (NET) Assay

#### 2.5.1. Neutrophil Isolation from Murine Bone Marrow

Neutrophils were isolated from BALB/c murine femur-derived bone marrow via the density gradient centrifugation method described by Swamydas and Lionakis [95]. Briefly, *n* = 3 mice were sacrificed via an isoflurane overdose and 6 femurs were collected. Serum-free RPMI 1640 cell culture medium was used to flush bone marrow out of the interior of each femur through a 100 µm cell strainer. Collected bone marrow was pooled, pelleted, and resuspended in ice-cold PBS, which was gently pipetted over a density column containing Histopaque 1119 overlayed with Histopaque 1077. After centrifugation at approximately 800× *g* for 30 min, without a break, neutrophils were collected at the Histopaque 1077–1119 interface, washed, and counted via a hemocytometer before plating.

#### 2.5.2. NET Assay Dosing Calculation

The dosing of the NPs for the NET assay was calculated based on the clinically relevant dose of current GBCAs such as MultiHance^®^ and Eovist^®^, where the dose of 0.05 mmol/kg of chelated construct [96,97] or 7.5 mg/kg Gd (*Dose_metal_*), is 50% or 100% of their currently used dose, respectively. Equation (2) shows how each dosing was calculated based on the metal content of each type of NP:

(2) $$Dosing\;\left(\frac{\mu g\;NP}{neutrophil}\right)=\frac{Dose_{metal}}{C_{neutrophil}*V_{blood}*NP_{metal}}$$

where *C_neutrophil_* is the average circulating neutrophil count from the female BALB/c mice at 8–16 weeks old (0.975 × 10^3^ neutrophil/µL [98]), *V_blood_* is the average total blood volume in a mouse (78.5 mL/kg [99,100]), and the *NP_metal_* is the metal content per mg of NP calculated from ICP-OES as described previously.

#### 2.5.3. Ex Vivo NET Assay

Isolated neutrophils were stained with CellTracker™ Deep Red (2 µM) to visualize the cell membrane and Hoescht 33342 (8.1 µM) to visualize the nuclei and cell-free DNA, according to manufacturer instructions. Neutrophils were plated at 15,000 cells/well in 96 well plates and then incubated with Gd-DTPA, bare MnO NPs, 3 NEMO particle formulations, bare Fe_3_O_4_ NPs, or 3 NEIO particle formulations for 3 h at 37 °C in HBSS. Metal oxide NPs/GBCA dosing was standardized based on the overall quantity of metal loading determined from ICP-OES; thus, each well received a dose of contrast agent containing 1 µg metal (Fe, Mn, or Gd) per 10,000 neutrophils, which is approximately equivalent to a clinically relevant whole-body dose of 7.5 mg/kg Gd. NEMO and NEIO particle formulations were as follows: bare metal oxide NPs and PLGA encapsulated metal oxide NPs with either 0%, 2.5%, or 5% (*w*/*w*) PLGA-PEG. Each group was plated in triplicate. Unstimulated neutrophils were used as a negative control; these cells only received 10 µL of PBS (vehicle) and did not receive NPs or Gd-DTPA. Cells were fixed for 15 min at room temperature in 4% paraformaldehyde. Twenty fields of view (FOVs) were randomly collected per well on an upright Nikon A1R confocal microscope with a 40× objective.

Neutrophils and NETs were characterized automatically using a custom recipe in Nikon’s NIS-Elements general analysis software based on thresholding, size, and morphology. For preprocessing, functions including contrast enhancement, dark background removal, AI-assisted denoising, and a median filter were applied uniformly across all FOVs for neutrophils and NETs. Binary masks for neutrophils were then generated by filtering CellTracker™ Deep Red-positive objects between 8 µm and 20 µm in diameter and between 0.35 and 1 in circularity, where 1 is a perfect circle. Each NET mask was defined by filtering Hoescht 33342-positive objects greater than 10 µm in length on their longest axes with circularities between 0 and 0.7. We further required that the areas of the DNA objects include CellTracker™ Deep Red-positive objects to be characterized as NETs to distinguish them as having originated from neutrophils. The total NET area per neutrophil was calculated for each FOV to account for FOVs with more or fewer neutrophils.

### 2.6. Characterization of Neutrophil dsDNA Release, Reactive Oxygen Species Production, and Cytokine Release

Neutrophils were plated at approximately 250,000 cells/well in 6 well plates and were subsequently incubated with Gd-DTPA or a previously mentioned NP formulation for 3 h at 37 °C in HBSS. Metal dosing was standardized as discussed above. Unstimulated neutrophils were once more used as a negative control. The supernatant was removed from each well, aliquoted, and stored at −80 °C prior to downstream characterization studies.

Upon thawing the supernatant, a Quant-iT™ PicoGreen™ dsDNA Assay Kit was used according to manufacturer instructions to measure the quantity of cell-free double-stranded DNA released by neutrophils as a product of apoptosis or NETosis. Characterization of neutrophil inflammatory response was performed by measuring ROS presence within the supernatant via OxiSelect™ In Vitro ROS/RNS Assay Kit as per manufacturer instructions. Finally, neutrophil cytokine release in response to contrast agent stimulation was characterized with Proteome Profiler Mouse Cytokine Array Kit, Panel A according to the manufacturer’s instructions.

### 2.7. Scanning Electron Microscopy of NETs

Fifty thousand neutrophils/well were plated on coverslips within a 24 well plate and stimulated with contrast agents as previously described. Metal dosing was standardized as discussed above. Slides were gently rinsed with PBS 3 times and subsequently fixed with 4% paraformaldehyde after 3 h of incubation. Samples were then prepared for SEM characterization by first fixing the samples with 1% OsO_4_ followed by a series of washing steps with 0.1 M PBS. OsO_4_ has been found to crosslink cell components, particularly lipids, protecting the integrity of biological materials that would otherwise be damaged during preparation and imaging [101,102,103]. Then, the samples were dehydrated in a graded series of alcohol (30, 50, 70, 90, and 100% ethanol) followed by HMDS and allowed to air dry in a chemical fume hood overnight. HMDS is very volatile and quickly facilitates sample drying, ensuring the proper replacement of water and avoiding any shrinkage or loss of structure [102,104]. Images of NET structures were acquired with a Hitachi Scanning Electron Microscope S4700 in UHR-A mode at 5 kV.

### 2.8. Neutrophil Metal Content

For metal content assessment, neutrophils were plated at approximately 250,000 cells/well in 6 well plates and were subsequently stimulated with contrast agents as described. Following incubation, wells were gently rinsed with PBS 3 times to remove free Gd-DTPA or NPs. After washing, the remaining neutrophils were digested with 0.5 mL of HCl trace metal grade. The resultant solution was run under EPA Method 200.8 Revision 5.4 on a PerkinElmer NexION 2000 ICP-mass spectrometer. All samples were preserved to pH ≤ 2 before analysis.

### 2.9. Statistical Analysis

Unless otherwise indicated, all data are expressed as mean ± standard error. Comparison between groups was conducted via ANOVA with a post hoc Holm correction. *p*-values less than 0.05 were considered significant.

## 3. Results

### 3.1. Synthesized MnO and Fe_3_O_4_ Bare NPs Displayed Small Sizes with Intended Oxidation State 

Two optimized batches for both types of metal oxide NPs (MnO and Fe_3_O_4_) were successfully fabricated via a thermal decomposition reaction. The physicochemical properties of all NP samples were determined including morphology, crystallinity, size, surface chemistry, and metal loading.

**Figure 1:**
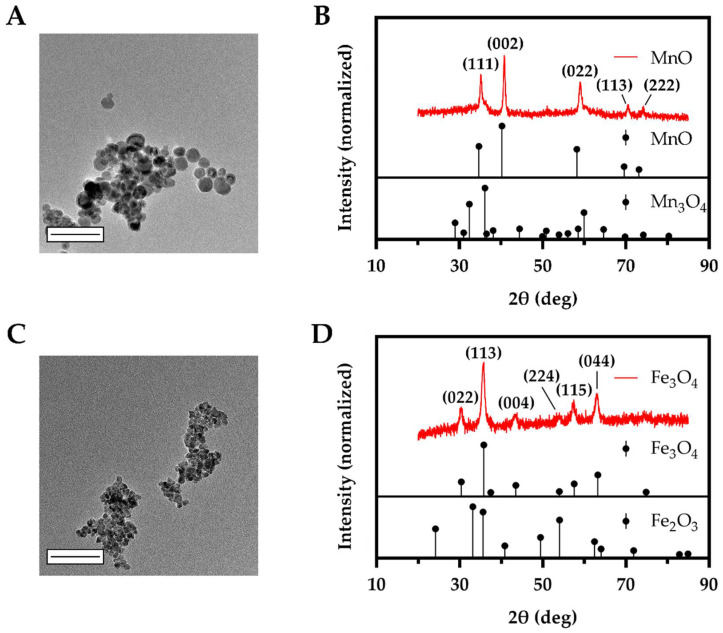
TEM images and XRD spectra of synthesized MnO (**A**,**B**) and Fe_3_O_4_ (**C**,**D**) bare NPs confirm the size and crystal structure. The measured XRD spectra of (**B**) MnO NPs and (**D**) Fe_3_O_4_ NPs for one of two batches synthesized are shown in red with the corresponding Miller indices shown for each peak. Standard diffraction peaks for known MnO, Mn_3_O_4_ (**B**) and Fe_3_O_4_, Fe_2_O_3_ (**D**) are shown in black using X’Pert HighScore. The measured spectra showed characteristic peaks of MnO and Fe_3_O_4_, respectively. Scale bars are 100 nm.

TEM was used to assess bare MnO and Fe_3_O_4_ NP size (Figure S1) and morphology (Figure 1A,C). The MnO NPs displayed a rounded octagonal shape, similar to that found in our previous study [19] and in the study by Nolis et al. [105]. The morphology of the Fe_3_O_4_ NPs was irregular but possessed a dominant triangular shape similar to that described by Belaïd et al. and Laurent et al. [106,107]. The average size according to TEM for bare MnO and Fe_3_O_4_ NPs was 22 ± 7 nm and 13 ± 4 nm, respectively.

XRD was used to evaluate the bare MnO and Fe_3_O_4_ NP crystal structure and bulk composition (Figure 1B,D and Figure S2) for all batches. All of the synthesized bare metal oxide NPs clearly displayed the highest known characteristic peaks for MnO and Fe_3_O_4_ obtained from X’Pert HighScore. Table 1 and Table S1 show the percent composition of MnO, Mn_3_O_4_, and Mn_2_O_3_ for two different batches of NPs, and Fe_3_O_4_ and Fe_2_O_3_ for two different batches of NPs. The intended oxidation state of either MnO or Fe_3_O_4_ was present as the highest percent composition for all NP formulations. Additionally, complementary techniques, HRTEM and SAED, corroborated the crystal structure of both metal oxide NPs (Figures S3 and S4). FTIR spectra of the bare NPs (Figure S5) were taken to confirm the oleylamine capping of the NPs and metal oxide bonds [19,87,108,109,110].

### 3.2. NEIO Particles Displayed Larger Particle Size and Aggregation While NEMO Particles Displayed Smaller and More Consistent Particle Size

MnO and Fe_3_O_4_ NPs were encapsulated via a single emulsion technique using PLGA with either 0%, 2.5%, or 5% (*w/w*) PLGA-PEG co-block polymer. The physicochemical properties of the NEMO and NEIO particles were determined, including morphology, size, surface chemistry, surface charge, PEGylation percentage, and metal loading.

**Figure 2:**
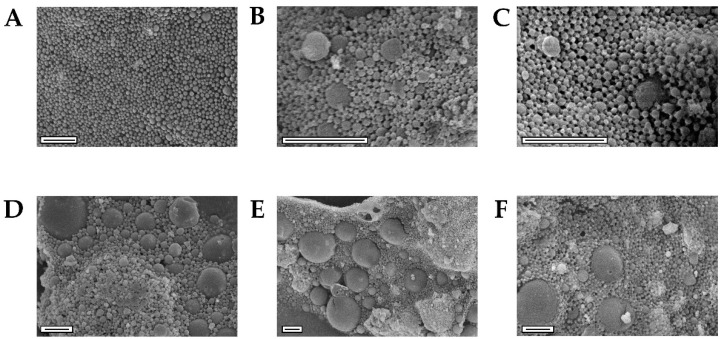
SEM images highlight the size uniformity of NEMO particles compared to NEIO particles. MnO NPs were encapsulated in PLGA with either (**A**) 0%, (**B**) 2.5%, or (**C**) 5% (*w*/*w*) PLGA-PEG. Similarly, Fe_3_O_4_ NPs were encapsulated in PLGA with either (**D**) 0%, (**E**) 2.5%, or (**F**) 5% PLGA-PEG. NEMO particles were observed to have a spherical shape with near uniform size distribution for all samples. NEIO particles were also observed to have a spherical shape but possessed a heterogenous size distribution for all samples. Scale bars are 1 µm.

To confirm successful polymer encapsulation of the metal oxide NPs, FTIR (Figure S6) was used to ensure that the characteristic PLGA peaks were present [111,112,113]. Encapsulation was further confirmed with SEM-EDS where both metals, manganese and iron, were only present where the polymer NPs were observed (Figures S7 and S8). NEMO and NEIO particles were also evaluated by SEM to assess morphology (Figure 2) and general dry size after encapsulation.

**Figure 3:**
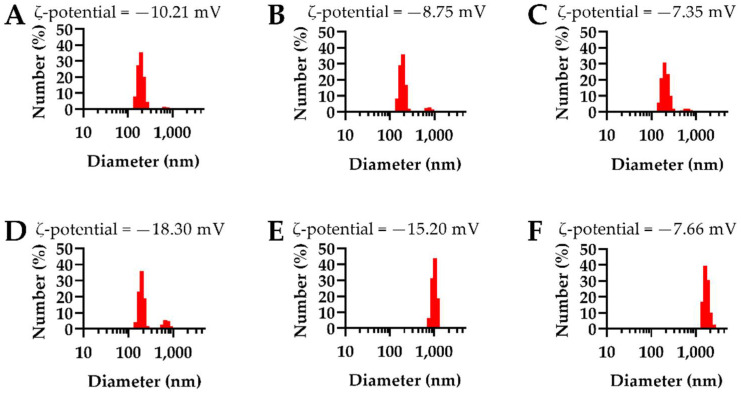
DLS and ζ-potentials reveal NEMO particle stability and NEIO particle aggregation with increasing PEGylation. Hydrodynamic size distributions and corresponding ζ-potentials are shown for (**A**–**C**) NEMO and (**D**–**F**) NEIO particles synthesized with increasing amounts of PLGA-PEG including (**A**,**D**) 0%, (**B**,**E**) 2.5%, and (**C**,**F**) 5% *w*/*w*. (**A**–**C**) NEMO particles had consistent distributions across all samples with ~94% or greater of NPs within the first, smaller-sized population leaving ~6% or less of NPs within the larger-sized population. (**D**) The size distribution of 0% PLGA-PEG NEIO particles was similar to their NEMO particle counterparts; however, ~16% of NPs lie within the larger-sized population, which is double compared to the NEMO particles. (**E**,**F**) For both 2.5% and 5% PLGA-PEG NEIO particles, the size distributions were monodispersed at greater than 1 µm. It should be noted that with increasing PEGylation, ζ-potential became more neutral for both NEMO and NEIO particle formulations.

All polymer encapsulated NPs exhibited spherical morphology; however, the NEMO particle size was more uniform compared to the NEIO particles, whose heterogeneity led to an increased overall dry size, as shown in Figure 2. To complement SEM, DLS was used to determine the hydrodynamic NP size distribution and the weighted average of the metal oxide NPs, as shown in Figure 3 and Table S2, respectively. 

For the NEMO particles, the hydrodynamic size populations were comparable to those found using SEM. The majority of particles were uniformly distributed and similar in size, with a small population of larger-sized particles for all three samples (Figure 3A–C). The 0% PLGA-PEG NEIO particles were comparable in size and distribution to the NEMO particles; however, as shown by DLS in Figure 3, the 2.5% and 5% PLGA-PEG NEIO particles had larger-sized populations. We hypothesize that these large DLS populations are due to NP aggregation post-synthesis from dipole-dipole interaction as described in the literature [114,115,116,117]. Additionally, the presence of the larger-sized population shown using SEM (Figure 2) could contribute to the larger peak seen using DLS for PEGylated NEIO particles. 

Aggregation is a common challenge to overcome in NP synthesis, as an ideal NP size for in vivo application is <200 nm to prevent vascular occlusion and increase cellular uptake [118,119,120,121,122]. There are several factors that can impact NP aggregation such as freeze-drying, the method of encapsulation, the surface charge, etc. [123,124,125]. Freeze-drying can cause mechanical stress and subsequent NP destabilization, and in this case, a cryoprotectant, such as trehalose, can be used to prevent aggregation [124,125]. As for the method of encapsulation, all variables were kept constant except for the type of metal oxide NP and the method of stirring. Due to the magnetic properties of Fe_3_O_4_ NPs, a non-ferrous, overhead stirrer was utilized in place of a magnetic stir plate during the solvent-evaporation step of the single emulsion technique. The overhead stirrer may have achieved different polymer and Fe_3_O_4_ NP mixing profiles than the conventional magnetic stir plate employed for the MnO polymer encapsulation process, resulting in large particles and aggregates as observed in all NEIO particle formulations using SEM and DLS (Figure 2 and Figure 3).

The surface charge of both NEMO and NEIO particles decreased with an increasing amount of PEGylation. Typically, more neutral charges lead to increased NP aggregation due to less interparticle electric repulsion forces; NEIO particles followed this trend and aggregated more as the ζ-potential came closer to zero, which may also be due to the dipole-dipole forces mentioned previously [114,115,116,117]. Interestingly, the NEMO particles did not tend to aggregate as the ζ-potential neutralized with PEGylation and instead maintained their stability. Additional studies will be required to uncover the mechanism behind these differences in aggregation between NEMO vs. NEIO particles. According to TGA (Figure S9), the actual PLGA-PEG percentage incorporated on the NP surface for 0%, 2.5%, and 5% PLGA-PEG for NEMO particles was 0%, 0.70%, and 1.1%, respectively, and for NEIO particles was 0%, 0.38%, and 0.77%, respectively. During the single emulsion technique, it is expected to see some loss of the PLGA-PEG, as any micelles formed with PLGA-PEG without metal oxide loading and any free-form polymer will be washed away during centrifugation. 

Table 2 shows the metal element loading and encapsulation efficiency (EE) of the different NPs used for the set of cell experiments shown below. Comparable results were observed during SEM-EDS analysis (Figures S7 and S8). The metal loading was applied to calculate the corresponding dosage of NPs that would be equivalent to a clinically relevant dose of GBCA (0.05 mmol/kg [96,97], or 7.5 mg/kg Gd).

### 3.3. Ex Vivo Fluorescent Imaging Reveals That NEMO Particles Elicit the Highest NETosis 

Neutrophils isolated from BALB/c mice were treated with standardized concentrations of Gd-DTPA, bare metal oxide NPs, or polymer encapsulated metal oxide NPs with varying levels of PEGylation. The NET formation was characterized using fluorescent staining; neutrophil supernatants were quantitatively evaluated for cell-free double-stranded DNA, ROS, and cytokine release. To confirm NET formation for each neutrophil, stimulated neutrophils were also imaged with SEM (Figure S10). 

**Figure 4:**
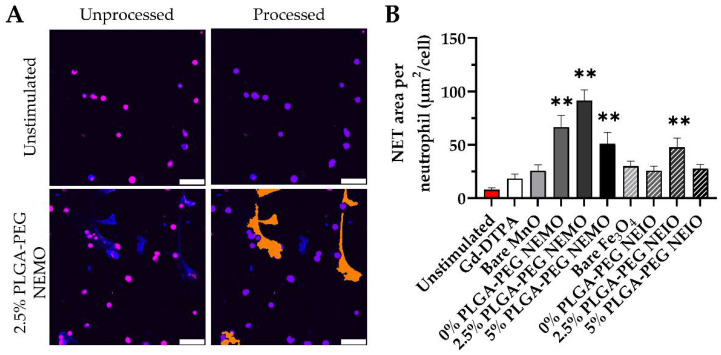
The fluorescence ex vivo NET assay demonstrates that NEMO particles elicit NETosis to a greater extent than NEIO particles. (**A**) Representative unprocessed and analyzed fluorescent ex vivo NET assay FOVs for unstimulated neutrophils and neutrophils stimulated with 2.5% PLGA-PEG NEMO particles are shown. The automated analysis of collected fluorescent NET assay images was utilized to define neutrophils and NETs in each FOV for every stimulation group. In the unprocessed images, Hoechst-33342 (DNA) staining is shown in blue, and CellTracker™ Deep Red (neutrophil) staining appears in pink. In the processed images, masks representing neutrophils are in purple and NETs in orange. Scale bar 50 µm. (**B**) The area of NETs per number of neutrophils was quantified and averaged for each stimulation group. The 2.5% PLGA-PEG NEMOs produced the most NETosis. ** indicates significance (*p* < 0.01). The error bars represent standard error.

The fluorescent NET assay illustrated significant differences in the level of NETs produced in response to the various NP treatments (Figure 4). Gd-DTPA, bare MnO, bare Fe_3_O_4_, 0% PLGA-PEG NEIO particles (PLGA only), and 5% PLGA-PEG NEIO particles all increased the level of NETosis relative to unstimulated neutrophils, albeit non-significantly. However, the 0–5% PLGA-PEG NEMO groups and the 2.5% PLGA-PEG NEIO groups all elicited a robust pro-NETotic response that was significantly greater than the control group. For both NEMO and NEIO particles, the 2.5% PLGA-PEG coating caused the greatest NET release, showing that NP surface chemistry modulates NET formation. Representative FOVs for all groups are available in Figure S11. We also observed that the 0–5% PLGA-PEG blank NPs (without metal oxide) did not appear to significantly enhance NETosis on their own (Figure S12).

### 3.4. MRI Contrast Agents Provoke Non-NETotic Cell-Free dsDNA Release, Extracellular ROS, and Altered Cytokine Expression

**Figure 5:**
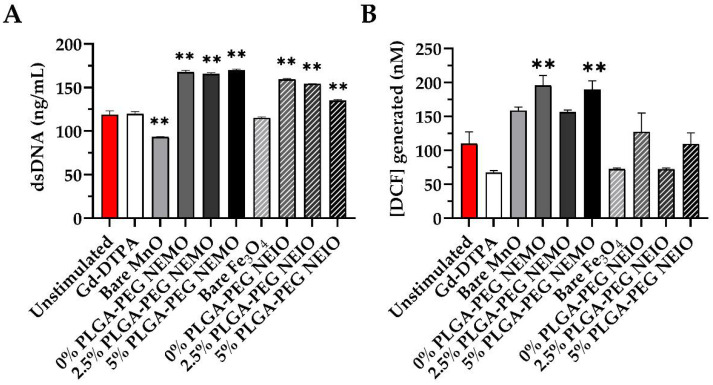
NEMO and NEIO particles both provoke neutrophil extracellular DNA release but only NEMO particles elevate ROS production. (**A**) Quant-iT ™ Picogreen ™ dsDNA assay was utilized to quantify the presence of cell-free dsDNA, which is indicative of NETosis, in each neutrophil sample’s supernatant following contrast agent exposure. dsDNA production was similar between the MnO and Fe_3_O_4_ NP formulations, despite significantly higher NETosis produced by MnO NPs. ** indicates significance (*p* < 0.01). The error bars represent standard error. (**B**) OxiSelect ™ In Vitro ROS/RNS assay was utilized to determine the relative difference in neutrophil oxidative stress in supernatants for each stimulation group; 2′, 7′-dichlorodihydrofluorescein (DCF) is the end product fluorescent indicator for total ROS/RNS levels in each sample. The 0% and 5% PLGA-PEG NEMO particles were the only groups to show enhanced relative ROS production. ** indicates significance (*p* < 0.01). The error bars represent standard error.

We sought to confirm the trends seen in our microscopy-based ex vivo NET assay by measuring the total amount of dsDNA released by neutrophils for each stimulation group (Figure 5a). Although dsDNA is not a perfect marker of NETosis, as it can also be present due to cell death, the results were similar to the trends observed for NETosis, with several interesting caveats. For instance, NEMO and NEIO formulations appeared to elicit similar amounts of cell-free dsDNA release. If NEIO-stimulated neutrophils provoke similar overall amounts of cell-free dsDNA release, but fewer NETs, this suggests that dsDNA may be released by a different mechanism such as apoptosis or necrosis. Additionally, NEIO formulations appeared to cause dsDNA release that decreased slightly, yet significantly with greater PEGylation; this was not the case for NEMO formulations. This suggests that higher PEGylation may reduce cytotoxicity specifically for NEIO formulations, resulting in lower non-NETotic dsDNA release. Interestingly, Gd-DTPA appeared to release more dsDNA than bare MnO and Fe_3_O_4_ NPs, whereas they produced fewer NETs. In fact, bare NPs appeared to repress dsDNA release compared to the other groups, though this was only significant for MnO NPs.

While NETs both mediate and indicate an inflammatory state, NETosis alone is not the sole aspect of neutrophil involvement in inflammation. In vivo, NETs both promote inflammation and occur as a direct result of it [126]. We hypothesized that increased NETosis would correlate to increased oxidative stress. Thus, we measured the presence of ROS in the neutrophil supernatant post-stimulation (Figure 5b). Again, it appeared that the MnO groups elicited the greatest oxidative response, more so than all Fe_3_O_4_ groups. However, only the 0% and 5% PLGA-PEG NEMO groups reached significance relative to the control. This suggests that NP surface chemistry and composition both modulate NETosis, oxidative stress, and dsDNA release. Different NP formulations may favor NETosis, ROS production, or possibly neutrophil cell death, which can also result in dsDNA release. Overall, this data supported the hypothesis that increased ROS production supported increased NETosis, as NEMO particles generally provoked more NETosis and ROS production than NEIO particles. However, the 2.5% PLGA-PEG NEMO and NEIO particles did not significantly enhance extracellular ROS production despite provoking the most robust NETosis. As the ROS assay was applied only to the supernatant, it is possible that the 2.5% PLGA-PEG NEMO and NEIO particles could have increased intracellular ROS compared to the other formulations. Gd-DTPA did not elicit increased NETosis or ROS production compared to the control; this further supports the assertion that Gd-DTPA does not directly impact neutrophil-mediated oxidative stress.

**Figure 6:**
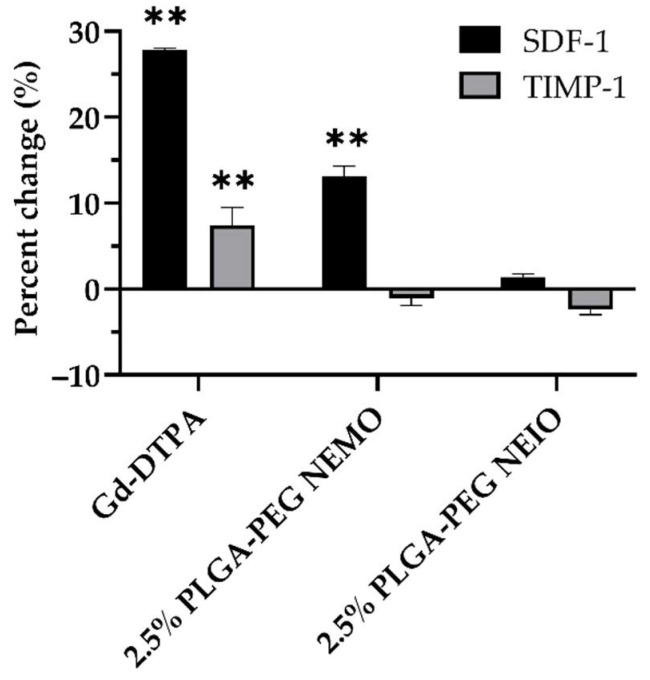
Gd-DTPA and NEMO particles alter neutrophil cytokine release. A Proteome Profiler Mouse Cytokine Array Kit, Panel A was utilized to investigate differences in neutrophil cytokine expression upon stimulation with different contrast agents. Changes in TIMP-1 and SDF-1 were expressed as percent change relative to unstimulated neutrophils. Gd-DTPA appeared to most significantly alter both SDF-1 and TIMP-1 production relative to unstimulated neutrophils. ** indicates significance (*p* < 0.01). Error bars represent standard error.

To determine whether the neutrophil response to MRI contrast agents had the capacity to influence other immune cells, we sought to examine whether the neutrophil cytokine response varied with different NP formulations (Figure 6). We decided to study the 2.5% PLGA-PEG NEMO and NEIO particle stimulated neutrophils, as these provoked the most NETosis relative to each formulation. We also assessed the neutrophil cytokine response to Gd-DTPA, a clinically relevant MRI contrast agent. Unstimulated neutrophils were utilized as a negative control. Although the relative presence of 40 different cytokines was measured via western dot blot membranes, only two showed significant differences in expression compared to the control in at least one group (Figure S13): Stromal cell-derived factor-1 (SDF-1; alternatively, CXCL12) and the tissue inhibitor of matrix metalloproteinase-1 (TIMP-1). SDF-1 is known to enhance neutrophil recruitment, and it is primarily expressed in the bone marrow, where it acts to retain neutrophils until they mature. Although not conventionally thought to be released by neutrophils themselves, some studies have illustrated that neutrophils do in fact produce SDF-1 at least at the mRNA level [127]. TIMP-1 is also known to elicit NETosis, particularly in the context of pancreatic cancer [128]. The production of each cytokine, but lack of increased NETosis, suggests that GBCAs have the ability to influence neutrophil chemoattraction. In vivo, this may result in NETosis due to influences from other cells such as platelets and the endothelium on recruited neutrophils.

### 3.5. MRI Contrast Agents Yield Differential Leukocyte Phagocytosis Dependent on Formulation 

**Figure 7:**
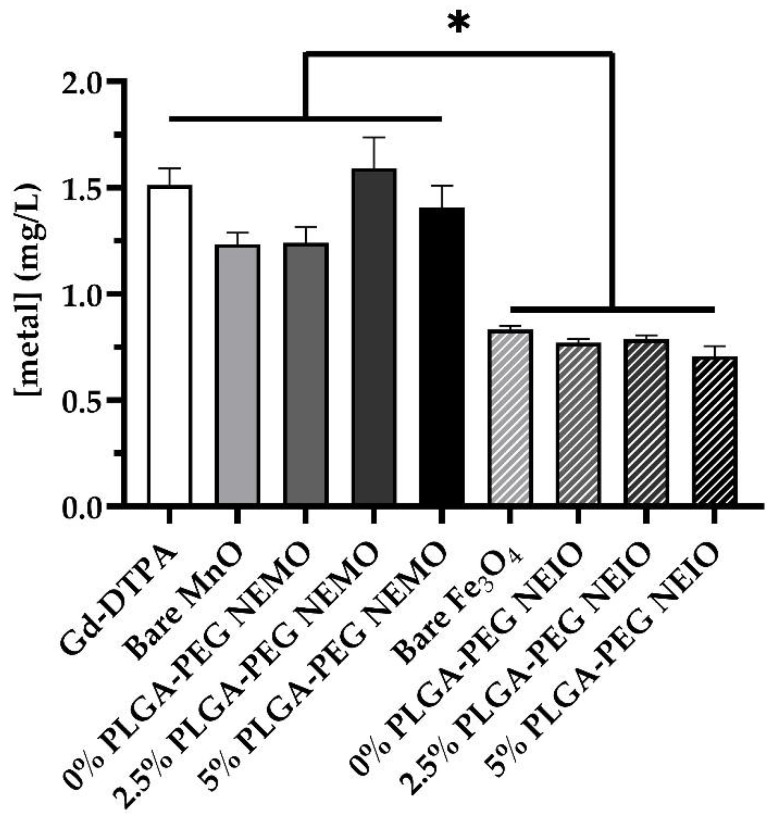
NEMO particles and Gd-DTPA are more readily internalized by neutrophils than NEIO particles. ICP-mass spectrometry was utilized to quantify the level of NP uptake by neutrophils for each stimulation group. The concentration of Gd, Mn, and Fe was measured in neutrophils treated with Gd-DTPA, bare MnO, the NEMO groups, bare Fe_3_O_4_, and the NEIO groups, respectively. Fe_3_O_4_ NP formulations appeared to be internalized at significantly lower rates than in the other groups. * indicates significance (*p* < 0.05). The error bars represent standard error.

Because we had observed significant increases in NETosis upon stimulation with NEMO particles specifically, we questioned whether this could be attributed to enhanced neutrophil uptake. As such, ICP-mass spectrometry was utilized to determine whether the differences in NETosis observed between various MRI contrast agent formulations corresponded with altered neutrophil phagocytosis (Figure 7). Indeed, internalization of all Fe_3_O_4_ formulations was significantly reduced compared to MnO and Gd-DTPA, suggesting that Fe_3_O_4_ may cause a smaller NETotic response simply because it is taken up less by neutrophils. Uptake, however, does not account for the differences seen between the groups within the metal oxide types, as these were not significantly different. Conversely, Gd-DTPA uptake appeared to be comparable to that of the MnO groups, but without pro-NETotic effects. Collectively, these results suggest that polymer coating, particularly PEG, may facilitate NETosis on its own in some capacity. Given the wide use of PEG in manufacturing NPs with biological applications, this merits further investigation.

## 4. Discussion

Because MRI contrast agents, particularly emerging NP-based contrast agents, may have the capacity to promote the formation of tumor-supporting NETs, we incubated murine neutrophils with Fe_3_O_4_ or MnO NPs encapsulated with or without PLGA or PLGA-PEG. We also stimulated neutrophils with Gd-DTPA to examine whether clinically utilized contrast agents altered neutrophil behavior. Our results suggest that MnO-based MRI contrast agents are more likely to elicit a pro-NETotic response than their Fe_3_O_4_ based counterparts. Our study suggests that the PEGylation percentage modulates NETosis, as both PEG-free particles and particles with 5% PLGA-PEG coating produced lower levels of NETosis than particles with 2.5% PLGA-PEG coating; in fact, there was no significant difference between the 0% PLGA-PEG and 5% PLGA-PEG NEIO particle-invoked NETosis compared to the control. PLGA encapsulation may also contribute to NETosis, as evidenced by bare MnO NPs causing substantially less NET formation despite similar uptake levels to the 0% PLGA-PEG NEMO particles. However, it is important to note that blank polymer NPs do not appear to significantly enhance NETosis on their own (Figure S12), at least at the percentage PEGylation levels studied. Instead, it appears that both polymer encapsulation and a metal oxide core must be present for NETosis to be enhanced. While polymer encapsulation does not appear to be necessary for metal oxide NP uptake in neutrophils, our data suggest that it is necessary for NETosis. Therefore, it is possible that NPs must reach a specific location within the cell, likely either the nucleus or mitochondria, which supply the DNA for NET fibers, in order to trigger NETosis. Collectively, these findings raise some concern about the safety of metal oxide NP MRI contrast agents in patients for which an increased inflammatory burden would be detrimental, as would be the case in cancer. It is notable that Gd-DTPA caused the smallest overall increase in NETosis relative to all other groups. Although the safety of GBCAs has been questioned due to heavy metal accumulation in the body, these concerns are not known to be directly linked to inflammation [129,130]. Our results support this assertion, as we did not observe elevated NETosis, DNA release, or ROS production in neutrophils stimulated with Gd-DTPA. As a chelate, rather than a polymer-encapsulated NP, Gd-DTPA likely benefits from enhanced stability compared to NEMO and NEIO particles, resulting in less direct interaction between metal ions and the intracellular environment; this may explain why Gd-DTPA had a lesser effect on neutrophil behavior in the majority of the studies conducted.

Although NEMO particles as a group elicited significantly more NET formation than NEIO particles, their overall capacity to cause neutrophils to release cell-free dsDNA was similar. Interestingly, the 2.5% PLGA-PEG NEMO and NEIO particles provoked similar overall dsDNA release relative to other surface chemistries despite provoking the most NETosis. One possibility is that groups exhibiting low NETosis, but high dsDNA release may actually be promoting necrosis more than NETosis. As discussed by Yousefi et al. [131], necrosis and NETosis both result in DNA release, but only NETs retain a distinctive, fibrous morphology. Necrotic DNA release forms a cloud-like structure [132]. Our morphology-based analysis of fluorescently labeled neutrophils and NETs does not characterize such structures as NETs (Figure S11). Therefore, dsDNA analysis alone would not accurately capture the nuances of how the degree of PEGylation differentially affects neutrophil NETosis and cell death. Additional studies characterizing neutrophil apoptosis and necrosis, as they relate to NETosis and the pathways that provoke it, are needed to further elucidate the underlying mechanisms promoting neutrophil response to contrast agents. 

The upregulation of TIMP-1 and SDF-1 in Gd-DTPA treated neutrophils, despite the apparently low potential to trigger NET formation, merits further investigation. SDF-1 is a chemoattractant for both leukocytes and lymphocytes; it plays a role in keeping neutrophils within the bone marrow and in recruiting neutrophils to other tissues. Interestingly, elevated levels of TIMP-1 have been shown to promote liver metastasis of colorectal cancer by increasing hepatic SDF-1 expression, thereby enhancing metastasis by attracting neutrophils to the liver pre-metastatic niche where they take on tumor-supporting phenotypes, presumably including NETosis [133]. Similar TIMP-1 mediated phenomena have been observed in pancreatic and breast cancer [128,134]. Neutrophils possess a matrix metalloproteinase (MMP) known as MMP9, which can form a non-inhibitory complex with TIMP-1, despite the latter being an MMP inhibitor [135]. While the functional significance of this complex is unclear, it is conceivable that it is functionally analogous to the interaction between TIMP-2 and MMP2, which ultimately results in the activation of MMP2 via other MMPs that subsequently interact with this complex [136]. If this is the case for the non-inhibitory TIMP-1/MMP9 complex, MMP9 activity would be enhanced. MMP9 specifically contributes to the pro-inflammatory, proteolytic functions of NETs [62]. Outside of MMP9 inhibition, TIMP-1 may contribute to other tumor-supporting phenomena including enhanced proliferation [137] and epithelial to mesenchymal transition [134]. Therefore, the fact that Gd-DTPA appears to upregulate these factors may be cause for concern. Although the increases in expression that we observed were modest, it is notable that they are occurring in neutrophils, which produce a relatively minor quantity of these cytokines compared to populations such as endothelial cells. If Gd-DTPA, or GBCAs in general, were to provoke similar effects systemically, there may be concerning subclinical consequences for cancer patients receiving MRI with contrast.

This is both the first study to specifically examine MnO NPs in relation to NETosis and the first study directly comparing leukocyte response between a clinically utilized GBCA and metal oxide NPs being developed as MRI contrast agents, as well as one of the few existing studies examining how modulating NP properties impacts NET release (Table 3). However, there are some limitations to this study that need to be further explored to better characterize both how metal oxide NPs and traditional MRI contrast agents affect leukocyte behavior, and whether this may promote poor outcomes, particularly for cancer patients. For instance, this study solely utilized isolated bone marrow-derived neutrophils divorced from a physiologic context; thus, it cannot account for the effects of corona formation from plasma proteins on NPs. The protein corona has the potential to alter NP uptake and subsequent neutrophil behavior [138]. Varying surface chemistries have the potential to alter this corona and therefore the overall consequences of the NP–neutrophil interaction [139]. Additionally, the use of healthy bone marrow-derived leukocytes precludes the ability to draw conclusions about peripheral neutrophils in tumor-bearing environments. As the presence of either a primary tumor or local metastatic lesions is known to prime neutrophils towards a pro-NETotic state in order to support metastasis, the observed effects in our study may be more pronounced in isolated neutrophils from tumor-bearing mice, and therefore of greater clinical concern [140]. Utilizing peripheral or tissue-resident neutrophils, specifically from common metastatic sites, isolated from tumor-bearing animals would address this. Examining a broader range of surface chemistries such as other polymers and functional groups in addition to NPs without metal oxide encapsulation would better enable conclusions regarding the effects of surface functionalization versus encapsulated metal content on leukocyte behavior. Finally, given that we did not attain similar size distributions for PEGylated NEMO and NEIO formulations, this study cannot fully account for size-mediated effects. The apparent difference in size seems to be partially mediated by the aggregation of Fe_3_O_4_ NPs; however, our results stand in contrast to Bilyy et al.’s [78] study which showed that aggregated Fe_3_O_4_ NPs contributed to enhanced NET formation in rabbit vasculature. Despite this, it is notable that the observed trend of NEMO particles enhancing NETosis to a greater extent than NEIO particles still held for the 0% PLGA-PEG formulations, as both the NEMOs and the NEIOs had comparable size distributions for this encapsulation. For instance, if size alone had prevented the 2.5% and 5% PLGA-PEG NEIO particles from enhancing NETosis to the levels produced by NEMO particles, then we would expect that the 0% PLGA-PEG NEIO treatment would have provoked comparable NETosis to NEMO groups; however, this was not the case. This supports the assertion that the presence of MnO-based NPs specifically caused greater NETosis than Fe_3_O_4_ based NPs.

## 5. Conclusions

Although further work is needed to better characterize the effects of MRI contrast agents on neutrophil phenotype in vivo, our work constitutes the first examination of the impact of MnO NPs and GBCAs on NETosis. Consideration of off-target cellular level effects is critical in NP contrast agent design to ensure that diagnostic imaging procedures do not negatively impact patient health. In this study, MnO NPs substantially increased neutrophil NETosis and the oxidative burden for specific surface chemistries, both of which could enhance the metastasis-supporting phenotype of neutrophils in the context of cancer. Meanwhile, our data support the hypothesis that Fe_3_O_4_ NPs may be safer than MnO NPs, as they produced less overall NETosis, extracellular ROS, cytokine release, and neutrophil uptake. However, the elevated free dsDNA release associated with Fe_3_O_4_ NP exposure could indicate an increase in neutrophil necrosis, which merits further study. Even though Gd-DTPA did not provoke NETosis, free dsDNA release, or ROS production, it was found to upregulate cytokines that are known to play a role in metastasis. Because all of our studies relate solely to the neutrophil response, much remains to be explored regarding the interactions between MRI contrast agents and other blood cells such as the endothelium, monocytes, circulating tumor cells, erythrocytes, and platelets. Our studies illustrate the critical importance of examining a broader range of cellular population responses to NPs throughout the design process to produce truly optimized NPs free of off-target effects.

## Figures and Tables

**Table 1:** Percent composition of the first synthesized batches of bare metal oxide NPs from X-Pert HighScore.

Metal Oxide Composition	MnO	Mn_3_O_4_	Mn_2_O_3_	Fe_3_O_4_	Fe_2_O_3_
MnO NPs	72%	21%	8%	-	-
Fe_3_O_4_ NPs	-	-	-	81%	19%

**Table 2:** Metal loading and EE percentage for each type of NP based on ICP-OES analysis.

Type of NP	Metal Loading (mg Metal Element/mg NP)		EE (%)
	Mn	Fe	
Bare MnO	0.63	-	-
0% PLGA-PEG NEMO	0.28	-	38
2.5% PLGA-PEG NEMO	0.23	-	51
5% PLGA-PEG NEMO	0.29	-	61
Bare Fe_3_O_4_	-	0.57	-
0% PLGA-PEG NEIO	-	0.22	69
2.5% PLGA-PEG NEIO	-	0.25	75
5% PLGA-PEG NEIO	-	0.32	21

**Table 3:** A summary of the literature examining how engineering NP characteristics impacts NETosis.

Type of NP	Study Parameter	Key Findings
Silver [77]	Concentration, size	↑ 5 nm [AgNP] ↑ NETosis ↑ 100 nm [AgNP] --- NETosis
Gold [83]	Size	↓ Size ↑ NETosis
Cationic liposomes [82]	Surface chemistry (cationic surfactants)	↑ Surface charge ↑ NETosis
Iron oxide [78]	Surface chemistry (lauric acid, dextran, or albumin)	NET/NP aggregation ↓ for dextran or albumin coated NPs
Manganese oxide and iron oxide *	Metal oxide, 0–5% PLGA-PEG encapsulation	NETosis --- for bare NPs NETosis ↑ for NEMO particles NETosis ↑ for 2.5% PLGA-PEG

↑ indicates increase, --- indicates no effect relative to control, ↓ indicates decrease, * indicates this study.

## Data Availability

All relevant data are within the manuscript and its Supporting Material.

## Supplementary Materials

The following supporting information can be downloaded at: https://www.mdpi.com/article/10.3390/bios12020123/s1, Figure S1. Size distributions of bare metal oxide NPs, Figure S2. XRD spectra confirm crystal structure of the second synthesized batches of bare metal oxide NPs, Table S1. Percent composition of the second synthesized batches of bare metal oxide NPs from X-Pert HighScore, Figure S3. High-Resolution TEM (HRTEM) and Selected Area Electron Diffraction (SAED) confirm the crystal structure of MnO NPs, Figure S4. HRTEM and SAED confirm the crystal structure of Fe_3_O_4_ NPs, Figure S5. FTIR spectra for bare metal oxide NPs confirm oleylamine surface coating, Figure S6. FTIR spectra for NEMO and NEIO particles confirm PLGA encapsulation, Figure S7. SEM-Energy Dispersive X-ray Spectroscopy (EDS) of NEMO particles verify the PLGA encapsulation, Figure S8. SEM-EDS of NEIO particles verify the PLGA encapsulation, Table S2. Average hydrodynamic size for NEMO and NEIO particles, Figure S9. ThermoGravimetric Analysis (TGA) for NP PEGylation quantification, Figure S10. SEM images reveal NETs produced by neutrophils exposed to contrast agents, Figure S11. Fluorescence ex vivo NET assay further confirms neutrophil NETotic response after incubation with contrast agents, Figure S12. Fluorescence ex vivo NET assay reveals that metal oxide-free (blank) PLGA-PEG NPs do not significantly enhance NETosis, Figure S13. Western dot blots show altered neutrophil cytokine release following contrast agent exposure. Reference [141] is cited in the supplementary materials.
