# Supplementary material for: PEGylation of Metal Oxide Nanoparticles Modulates Neutrophil Extracellular Trap Formation
Source: Biosensors (Basel). 2022 Feb 16;12(2):123. doi: 10.3390/bios12020123 (PMC8869785; doi:10.3390/bios12020123)
Supplement: Supplementary file 1 [file biosensors-12-00123-s001.zip › biosensors-1538526 SP-for publish.pdf]

biosensors

MDPI

Supplementary Information

PEGylation of Metal Oxide Nanoparticles Modulates Neutrophil Extracellular Trap Formation

Hunter T. Snoderly, Kasey A. Freshwater, Celia Martinez de la Torre, Dhruvi M. Panchal, Jenna N. Vito and Margaret F. Bennewitz *

Department of Chemical and Biomedical Engineering, West Virginia University, Morgantown, WV 26506, USA; htsnoderly@mix.wvu.edu (H.T.S.); kaf0005@mix.wvu.edu (K.A.F.); cemartinezdelatorre@mix.wvu.edu (C.M.d.l.T.); dp00016@mix.wvu.edu (D.M.P.); jnv0006@mix.wvu.edu (J.N.V.)
* Correspondence: margaret.bennewitz@mail.wvu.edu

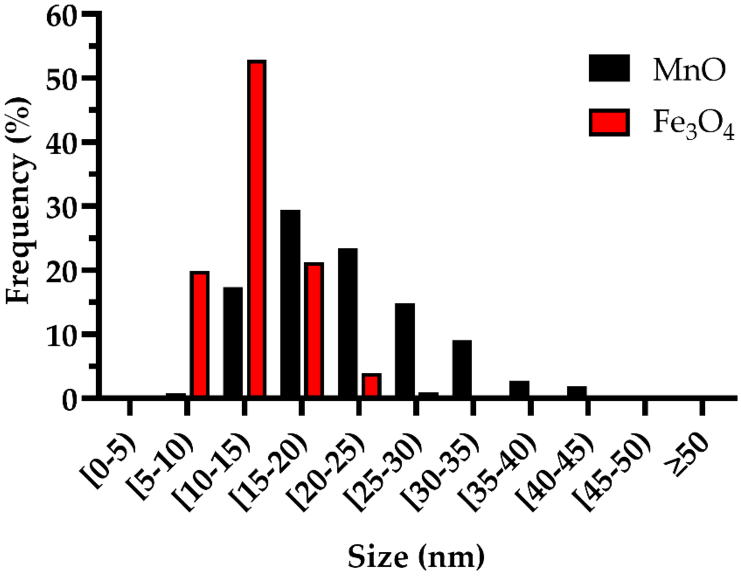

Figure S1. Size distributions of bare metal oxide NPs. Dry-size diameters for bare MnO (black) and bare Fe_3O_4 (red) NPs obtained from transmission electron microsopy (TEM, **Figure 1A,C**) using ImageJ software. MnO NPs have a wider distribution with an average size of 22 ± 7 nm; whereas, Fe_3O_4 NPs have a smaller distribution with an average size of 13 ± 4 nm.

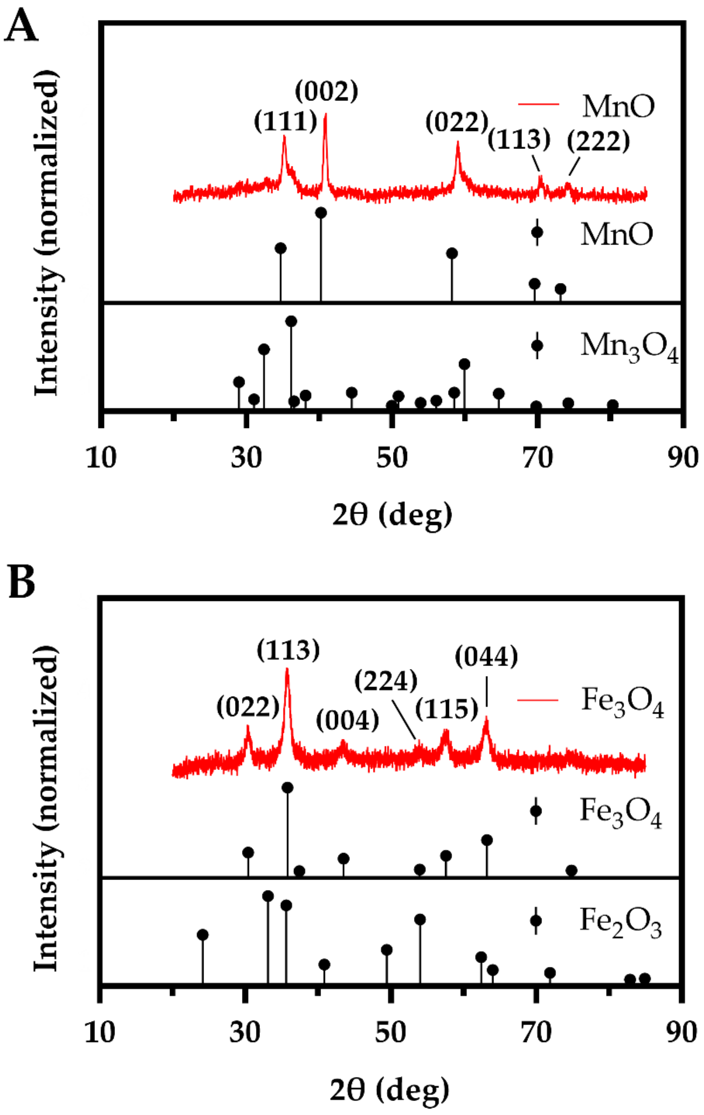

Figure S2. XRD spectra confirm crystal structure of the second synthesized batches of bare metal oxide NPs. Measured XRD spectra for (A) MnO NPs and (B) Fe_3O_4 NPs are shown in red with the corresponding Miller indices shown for each peak. Standard diffraction peaks for known (A) MnO, Mn_3O_4, and (B) Fe_3O_4, Fe_2O_3 from X'Pert HighScore are shown in black. The measured spectra showed characteristic peaks of MnO and Fe_3O_4, respectively [1,2].

Table S1. Percent composition of the second synthesized batches of bare metal oxide NPs from X-Pert HighScore. The percent composition of MnO, Mn_3O_4, and Mn_2O_3 for manganese oxide NPs, and Fe_3O_4 and Fe_2O_3 for iron oxide NPs are shown. The intended oxidation state of either MnO or Fe_3O_4 was present as the highest percent composition for all synthesized NPs.

Metal Oxide Composition	MnO	Mn_3O_4	Mn_2O_3	Fe_3O_4	Fe_2O_3
MnO NPs	66%	29%	5%	-	-
Fe_3O_4 NPs	-	-	-	78%	22%

A

B

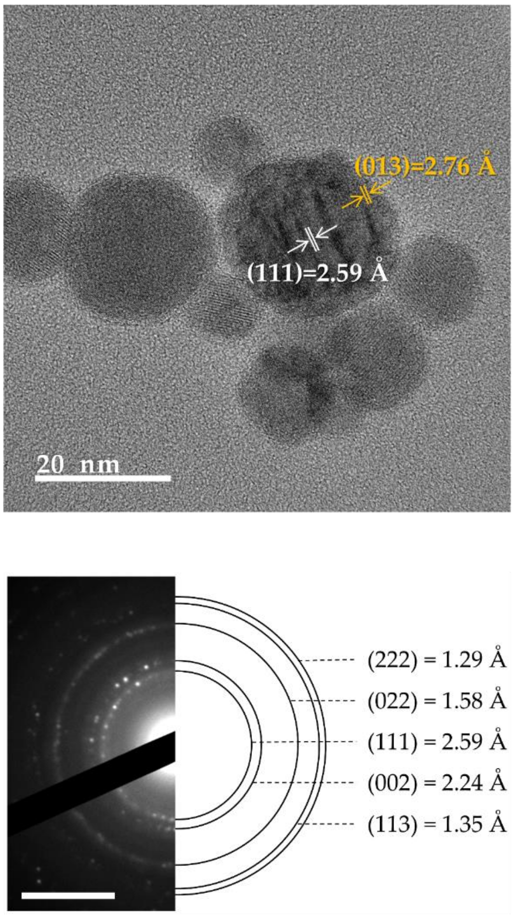

Figure S3. High-Resolution TEM (HRTEM) and Selected Area Electron Diffraction (SAED) confirm the crystal structure of MnO NPs. (A) Analysis of HRTEM images shows the main core formed by MnO (shown in white) and an outer layer of Mn_3O_4 (shown in yellow) corresponding to the oxidation of the most outer layer of NPs due to air contact, which agrees with our previous study and the literature [1,3]. (B) SAED showed the top five crystal planes agreeing with the XRD spectra shown in Figure 1B and Figure S2A. Scale bar in (B) is 5 nm^{-1}.

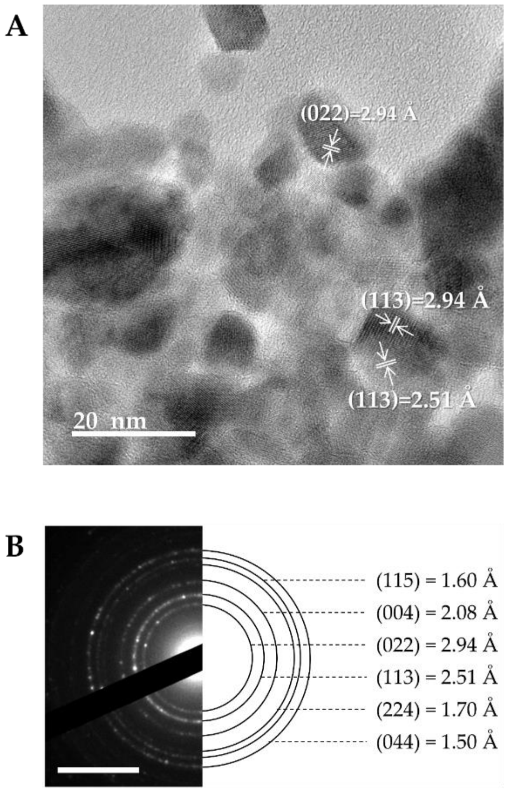

Figure S4. HRTEM and SAED confirm the crystal structure of Fe_3O_4 NPs. (A) Analysis of HRTEM images corroborated the crystal structure and phase of iron oxide NPs. The top two crystal planes, (022) and (113), shown in white, were found on several of the NPs. (B) SAED showed the top six crystal planes agreeing with the XRD spectra shown in **Figure 1D** and **Figure S2B**. Scale bar in (B) is 5 nm^{-1}.

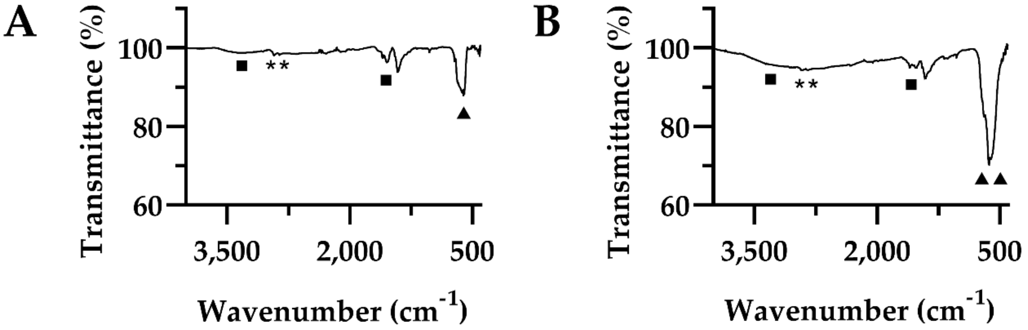

Figure S5. FTIR spectra for bare metal oxide NPs confirm oleylamine surface coating. For both spectra, peaks representing oleyl and amine groups indicate successful oleylamine NP capping from the thermal decomposition of either (A) $Mn(AcAc)_2$ or (B) $Fe(AcAc)_3$ in a 1:5 volume ratio of oleylamine and dibenzyl ether. Oleyl groups (∗) are present at 2850-2854 cm^{-1} (symmetric stretching of CH_2) and 2918-2926 cm^{-1} (asymmetric stretching of CH_2), and amine groups (■) are present at ~ 1593 cm^{-1} (symmetric stretching of NH_2) and 3300 cm^{-1} (asymmetric stretching of NH_2) [1,4,5]. (A) For bare MnO, peaks (▲) around 600 cm^{-1} represent the stretching vibration of Mn-O and Mn-O-Mn bonds indicating successful synthesis of MnO, which corroborates XRD analysis [1,6]. (B) For bare Fe_3O_4, spinel structure peaks (▲▲) between 400 cm^{-1} and 600 cm^{-1} represent Fe-O deformation in the octahedral and tetrahedral sites indicating successful synthesis of Fe_3O_4, which corroborates XRD analysis as well [2].

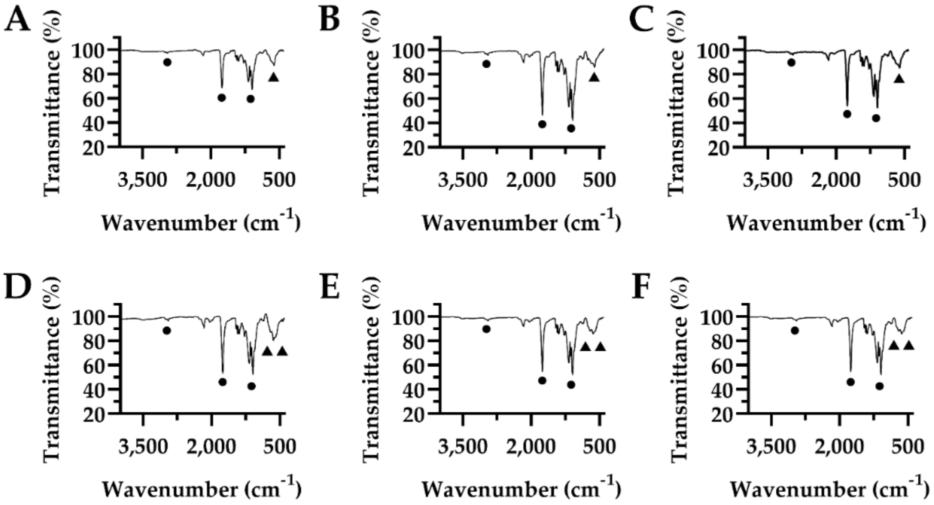

Figure S6. FTIR spectra for NEMO and NEIO particles confirm PLGA encapsulation. FTIR spectra are shown for PLGA NEMO particles with (A) 0%, (B) 2.5%, and (C) 5% PLGA-PEG and for PLGA NEIO particles with (D) 0%, (E) 2.5%, and (F) 5% PLGA-PEG. Characteristic PLGA peaks (●) are shown for all samples. The peaks at 2993 cm^{-1} and 2989 cm^{-1} represent the C–H stretch of CH_2 and –C–H–, respectively. The C=O stretching vibration of the ester bond is present at 1751 cm^{-1} and the C-O stretching is present at 1165 - 1087 cm^{-1}. [7–9] The shallow peaks present between 400 to 600 cm^{-1} represent the encapsulated MnO (▲) or Fe_3O_4 (▲▲), refer to **Figure S5**.

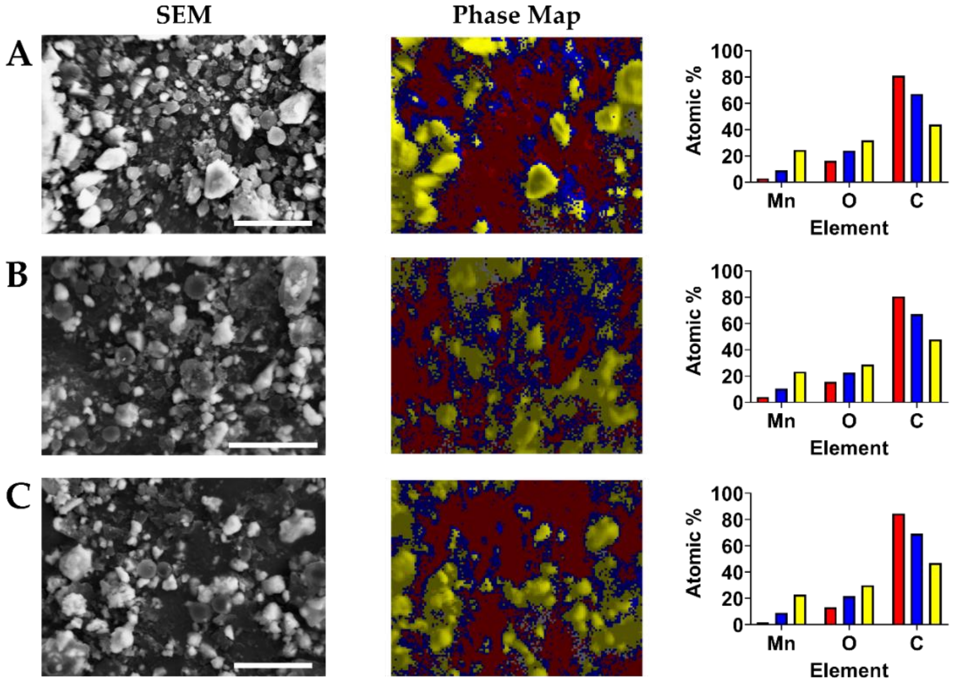

Figure S7. SEM-Energy Dispersive X-ray Spectroscopy (EDS) of NEMO particles verify the PLGA encapsulation. SEM and phase map images of PLGA NEMO particles with (A) 0%, (B) 2.5%, and (C) 5% PLGA-PEG. In all formulations, the highest manganese percentage (shown in yellow) was found to be localized where the NPs were in the SEM images; carbon originating from the polymer encapsulation was also localized along with manganese and oxygen (yellow). The carbon tape (shown in red) was mainly composed of carbon as expected. On the other hand, the blue region is the border of the NPs in contact with the carbon tape, which has an intermediate amount of manganese and oxygen. Scale bars 10 µm.

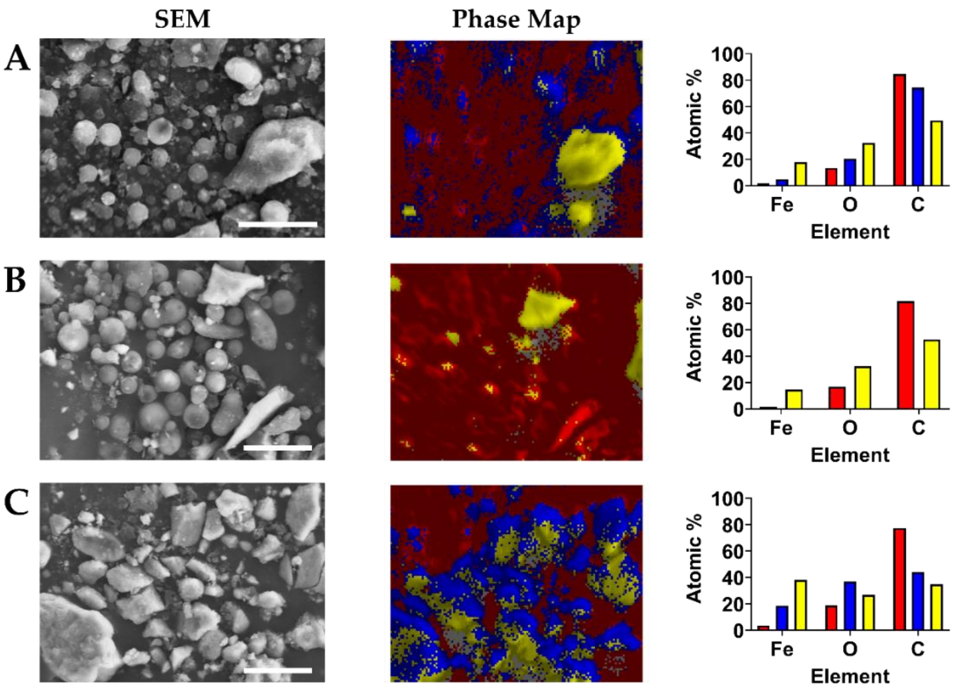

Figure S8. SEM-EDS of NEIO particles verify the PLGA encapsulation. SEM and phase map images of PLGA NEIO particles with (A) 0%, (B) 2.5%, and (C) 5% PLGA-PEG. In all formulations, the highest iron percentage (shown in yellow) was found to be localized where the NPs were in the SEM images; carbon originating from the polymer encapsulation was also localized along with iron and oxygen (yellow). The carbon tape (shown in red) was mainly composed of carbon as expected. In the case of (B) 2.5% PLGA-PEG, only the iron-encapsulated particles (yellow) and the main carbon region (red) were found. On the other phase maps (A) and (C), an intermediate region was found marked in blue where the border of the NPs meets the carbon tape**.** Scale bars 10 μm.

Table S2. Average hydrodynamic size for NEMO and NEIO particles. Weighted averages ($\bar{x}$) and deviations ($\pm\sigma$) for NP diameter were determined by DLS for 0%, 2.5%, and 5% PLGA-PEG NEMO and NEIO particles. All NEMO particles and 0% PLGA-PEG NEIO particles had a uniform distribution with a small, larger-sized population comparable to SEM (**Figure 2**). 2.5% and 5% PLGA-PEG NEIO particles had a single larger-sized population, which is attributed to NP aggregation; smaller-sized particles were present in SEM in addition to a larger-sized population.

% PLGA-PEG	Weighted Average (nm)			
	NEMO		NEIO	
	$\bar{x}$	$\pm\sigma$	$\bar{x}$	$\pm\sigma$
0%	217	15	276	16
2.5%	236	14	1008	42
5%	234	14	1764	50

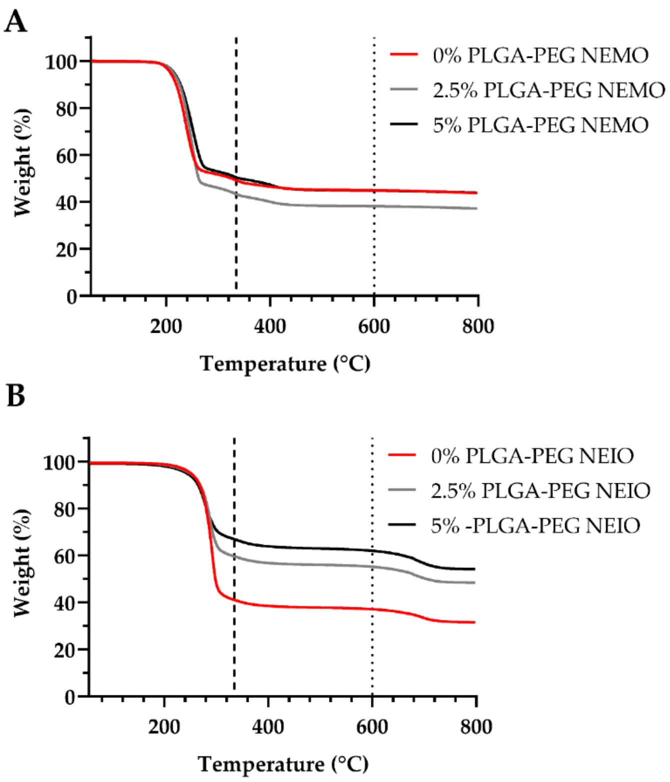

Figure S9. ThermoGravimetric Analysis (TGA) for NP PEGylation quantification. Percent (%) weight loss versus temperature (^{0}C) for (A) NEMO and (B) NEIO particles with 0% (red), 2.5% (grey), and 5% (black) PLGA-PEG added during single emulsion technique. Thermal degradation of PLGA is observed between 205^{0}C and 280^{0}C [10,11]. The dashed, vertical black line at 334.4^{0}C [12] and the dotted, vertical grey line at 600^{0}C represent the start of PLGA-PEG degradation and the end of degradation (kept consistent for all samples), respectively. The corresponding weight loss between these two vertical lines for each sample was used within a grafting density equation (**Equation 1**) [13,14] to determine PEGylation percentage via MATLAB®. The calculated NP PEGylation was 0%, 0.70%, and 1.1% for 0%, 2.5%, and 5% PLGA-PEG NEMO particles and was 0%, 0.38%, and 0.77% for 0%, 2.5%, and 5% PLGA-PEG NEIO particles. Note, the plateau region for each sample can vary due to different encapsulation efficiency (refer to **Table 2**).

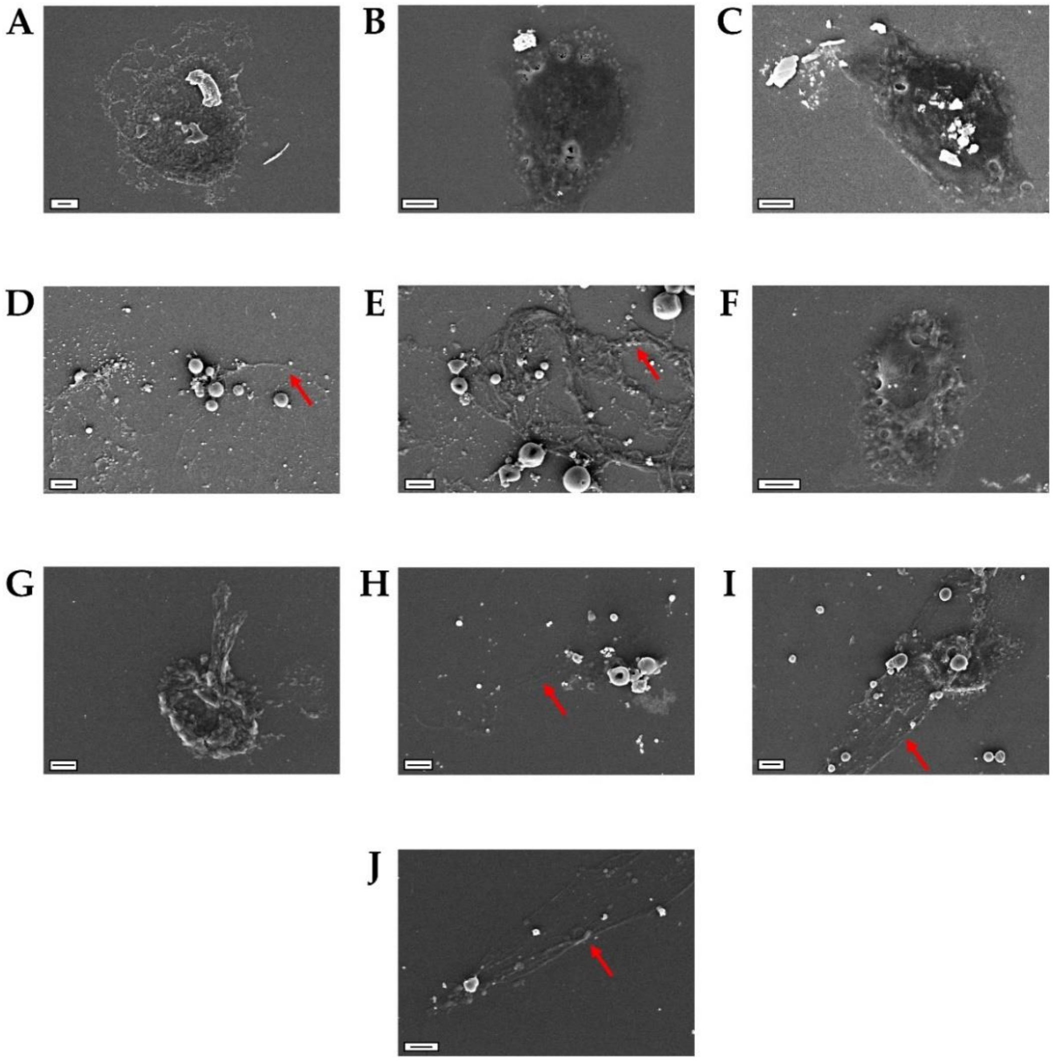

Figure S10. SEM images reveal NETs produced by neutrophils exposed to contrast agents. Representative SEM images highlighted the presence or lack of NETs produced by (A) unstimulated neutrophils, (B) Gd-DTPA stimulated neutrophils, (C) Bare MnO, (D) 0% PLGA-PEG NEMO, (E) 2.5% PLGA-PEG NEMO, (F) 5% PLGA-PEG NEMO, (G) Bare Fe_3O_4, (H) 0% PLGA-PEG NEIO, (I) 2.5% PLGA-PEG NEIO, and (J) 5% PLGA-PEG NEIO. Not all groups showed evidence of NETosis via SEM; for instance, NETs were not observed via SEM in most groups that appeared to have a less robust NETotic response as observed via ex vivo NET fluorescence assay (A, B, C, F, G). Observation of NETs via SEM could have been limited due to the overall small number of collected FOVs (around 11 per group). Red arrows illustrate examples of fibrous NET structures where applicable. Scale bars 2 μm.

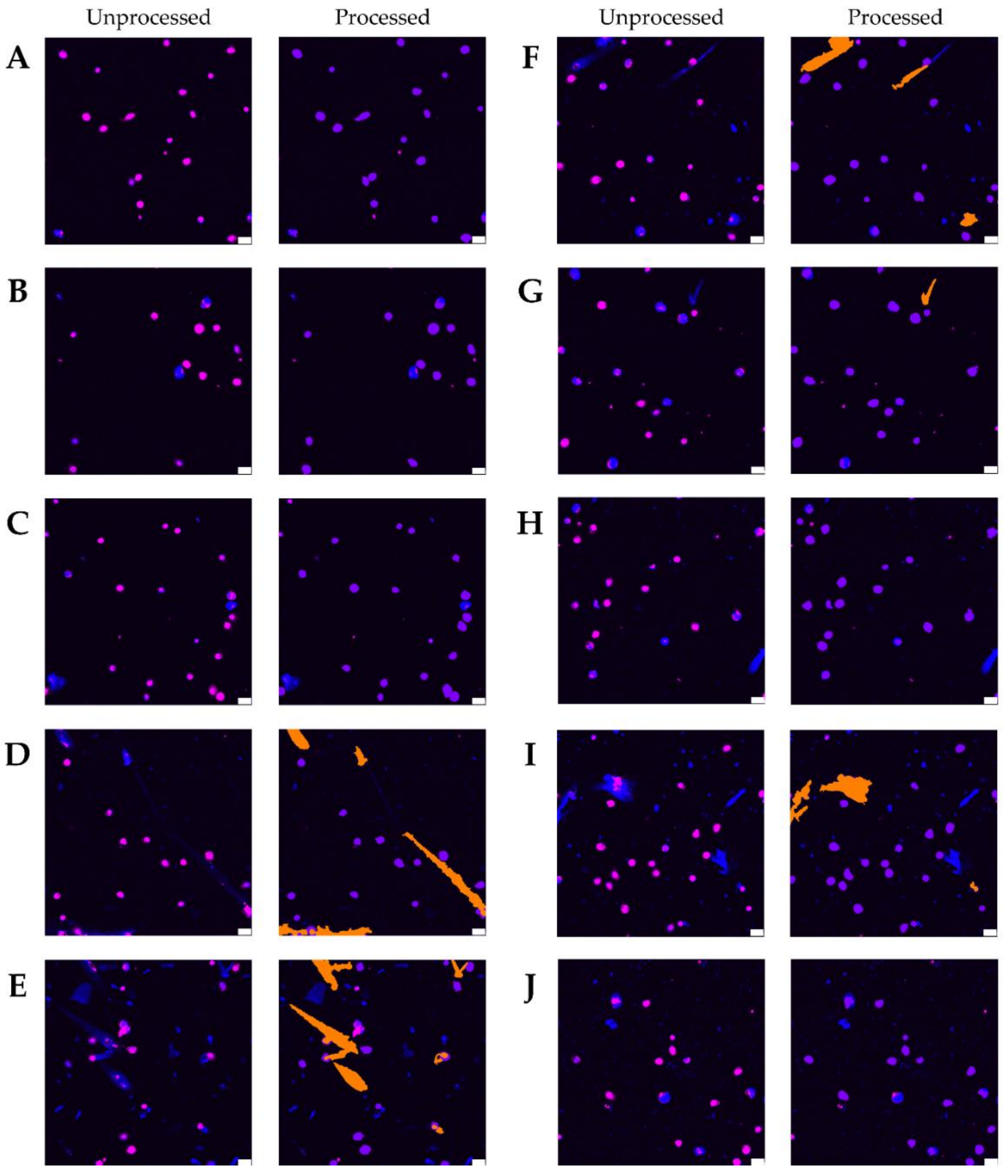

Figure S11. Fluorescence ex vivo NET assay further confirms neutrophil NETotic response after incubation with contrast agents. Representative unprocessed and analyzed fluorescent confocal ex vivo NET assay FOVs for (A) unstimulated neutrophils and neutrophils treated with (B) Gd-DTPA, (C) Bare MnO, (D) 0% PLGA-PEG NEMO, (E) 2.5% PLGA-PEG NEMO, (F) 5% PLGA-PEG NEMO, (G) Bare Fe_3O_4, (H) 0% PLGA-PEG NEIO, (I) 2.5% PLGA-PEG NEIO, and (J) 5% PLGA-PEG NEIO. Nikon Elements General Analysis software was utilized to automatically define neutrophils and NETs in each FOV for every stimulation group. In unprocessed images, Hoechst-33342 (DNA) staining is shown in blue and CellTracker™ Deep Red (neutrophil) staining in pink. In processed images, masks representing neutrophils are in purple and NETs in orange. Utilization of NET area enabled relative comparisons of overall pro-NETotitc response to stimuli. Note the stronger NET release from NEMO particles vs. NEIO particles. Scale bars 20 μm.

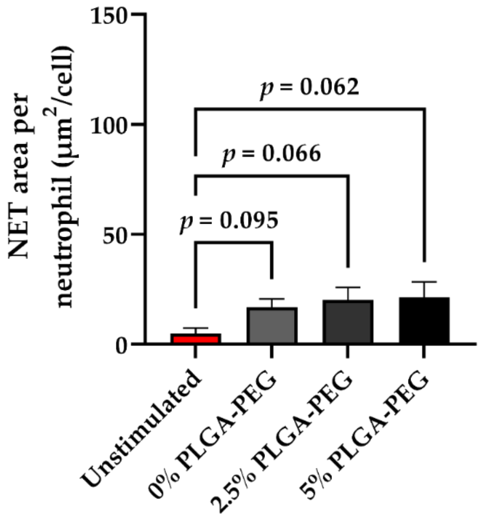

Figure S12. Fluorescence ex vivo NET assay reveals that metal oxide-free (blank) PLGA-PEG NPs do not significantly enhance NETosis. Neutrophils were left unstimulated or incubated with 0-5% blank PLGA-PEG NPs. NET assay and analysis were performed as previously described, with neutrophils from n=3 donor mice plated in triplicate for each group. 15 FOVs were collected per well. Area of NETs per number of neutrophils were quantified and averaged for each stimulation group. Although PLGA-PEG treatment slightly increased NETosis, no significance was detected. Error bars represent standard error.

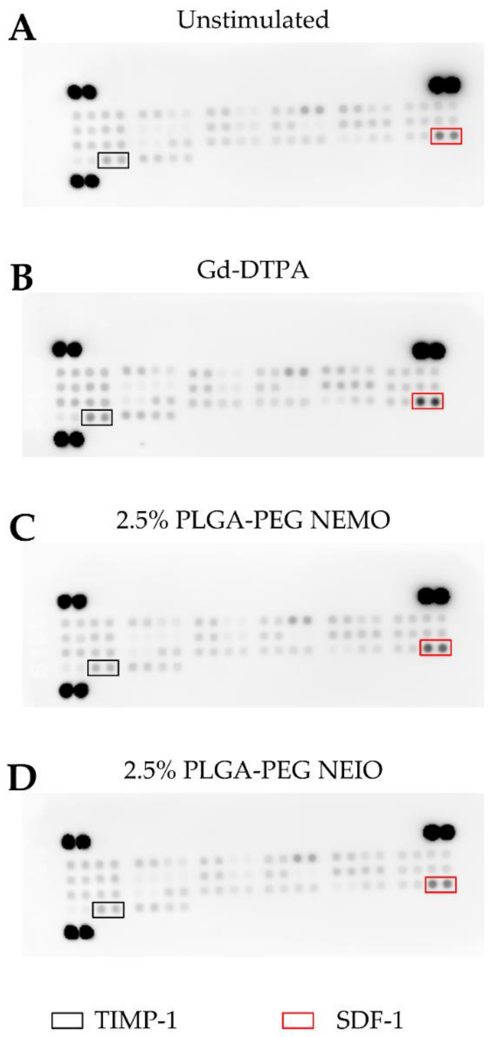

Figure S13. Western dot blots show altered neutrophil cytokine release following contrast agent exposure. Proteome Profiler Mouse Cytokine Array Kit, Panel A nitrocellulose membranes were utilized to assess neutrophil inflammatory response to contrast agents. Each dot blot is spotted with antibodies for 40 different cytokine targets. Compared treatment groups included (A) unstimulated neutrophils, (B) Gd-DTPA, (C) 2.5% PLGA-PEG NEMO, and (D) 2.5% PLGA-PEG NEIO. While expression was observed for several cytokines, only TIMP-1 (black box) and SDF-1 (red box) showed changes in expression from unstimulated neutrophils, with Gd-DTPA having the highest expression for both cytokines (Refer to **Figure 6**).

References

1. Martinez de la Torre, C.; Grossman, J.H.; Bobko, A.A.; Bennewitz, M.F. Tuning the Size and Composition of Manganese Oxide Nanoparticles through Varying Temperature Ramp and Aging Time. *Plos one* **2020**, *15*, e0239034.
2. Xu, Z.; Shen, C.; Hou, Y.; Gao, H.; Sun, S. Oleylamine as Both Reducing Agent and Stabilizer in a Facile Synthesis of Magnetite Nanoparticles. *Chem. Mater.* **2009**, *21*, 1778–1780, doi:10.1021/cm802978z.
3. Antoni, H.; Xia, W.; Masa, J.; Schuhmann, W.; Muhler, M. Tuning the Oxidation State of Manganese Oxide Nanoparticles on Oxygen- and Nitrogen-Functionalized Carbon Nanotubes for the Electrocatalytic Oxygen Evolution Reaction. *Phys. Chem. Chem. Phys.* **2017**, *19*, 18434–18442, doi:10.1039/C7CP02717F.
4. Perez De Berti, I.O.; Cagnoli, M.V.; Pecchi, G.; Alessandrini, J.L.; Stewart, S.J.; Bengoa, J.F.; Marchetti, S.G. Alternative Low-Cost Approach to the Synthesis of Magnetic Iron Oxide Nanoparticles by Thermal Decomposition of Organic Precursors. *Nanotechnology* **2013**, *24*, 175601, doi:10.1088/0957-4484/24/17/175601.

5. Mourdikoudis, S.; Liz-Marzán, L.M. Oleylamine in Nanoparticle Synthesis. *Chem. Mater.* **2013**, *25*, 1465–1476, doi:10.1021/cm4000476.

6. Zheng, M.; Zhang, H.; Gong, X.; Xu, R.; Xiao, Y.; Dong, H.; Liu, X.; Liu, Y. A Simple Additive-Free Approach for the Synthesis of Uniform Manganese Monoxide Nanorods with Large Specific Surface Area. *Nanoscale Res Lett* **2013**, *8*, 166, doi:10.1186/1556-276X-8-166.

7. Wang, H.; Zhao, Y.; Wu, Y.; Hu, Y.; Nan, K.; Nie, G.; Chen, H. Enhanced Anti-Tumor Efficacy by Co-Delivery of Doxorubicin and Paclitaxel with Amphiphilic Methoxy PEG-PLGA Copolymer Nanoparticles. *Biomaterials* **2011**, *32*, 8281–8290, doi:10.1016/j.biomaterials.2011.07.032.

8. Arasoglu, T.; Derman, S.; Mansuroglu, B. Comparative Evaluation of Antibacterial Activity of Caffeic Acid Phenethyl Ester and PLGA Nanoparticle Formulation by Different Methods. *Nanotechnology* **2016**, *27*, 025103, doi:10.1088/0957-4484/27/2/025103.

9. Jiang, P.; Yu, D.; Zhang, W.; Mao, Z.; Gao, C. Influence of Bovine Serum Albumin Coated Poly(Lactic-Co-Glycolic Acid) Particles on Differentiation of Mesenchymal Stem Cells. *RSC Adv.* **2015**, *5*, 40924–40931, doi:10.1039/C5RA07219K.

10. Bennewitz, M.F.; Lobo, T.L.; Nkansah, M.K.; Ulas, G.; Brudvig, G.W.; Shapiro, E.M. Biocompatible and PH-Sensitive PLGA Encapsulated MnO Nanocrystals for Molecular and Cellular MRI. *ACS Nano* **2011**, *5*, 3438–3446, doi:10.1021/nn1019779.

11. Silva, M.F.; Hechenleitner, A.A.W.; Irache, J.M.; Oliveira, A.J.A. de; Pineda, E.A.G. Study of Thermal Degradation of PLGA, PLGA Nanospheres and PLGA/Maghemite Superparamagnetic Nanospheres. *Mat. Res.* **2015**, *18*, 1400–1406, doi:10.1590/1516-1439.045415.

12. Jusu, S.M.; Obayemi, J.D.; Salifu, A.A.; Nwazojie, C.C.; Uzonwanne, V.; Odusanya, O.S.; Soboyejo, W.O. Drug-Encapsulated Blend of PLGA-PEG Microspheres: In Vitro and in Vivo Study of the Effects of Localized/Targeted Drug Delivery on the Treatment of Triple-Negative Breast Cancer. *Sci Rep* **2020**, *10*, 14188, doi:10.1038/s41598-020-71129-0.

13. Benoit, D.N.; Zhu, H.; Lilierose, M.H.; Verm, R.A.; Ali, N.; Morrison, A.N.; Fortner, J.D.; Avendano, C.; Colvin, V.L. Measuring the Grafting Density of Nanoparticles in Solution by Analytical Ultracentrifugation and Total Organic Carbon Analysis. *Anal. Chem.* **2012**, *84*, 9238–9245, doi:10.1021/ac301980a.

14. Adib, A.A.; Nazemidashtarjandi, S.; Kelly, A.; Kruse, A.; Cimatu, K.; David, A.E.; Farnoud, A.M. Engineered Silica Nanoparticles Interact Differently with Lipid Monolayers Compared to Lipid Bilayers. *Environ. Sci.: Nano* **2018**, *5*, 289–303, doi:10.1039/C7EN00685C.
